# Expression of heavy chain‐only antibodies can support B‐cell development in light chain knockout chickens

**DOI:** 10.1002/eji.201546171

**Published:** 2016-08-02

**Authors:** Benjamin Schusser, Ellen J. Collarini, Darlene Pedersen, Henry Yi, Kathryn Ching, Shelley Izquierdo, Theresa Thoma, Sarah Lettmann, Bernd Kaspers, Robert J. Etches, Marie‐Cecile van de Lavoir, William Harriman, Philip A. Leighton

**Affiliations:** ^1^ Reproductive BiotechnologyTechnische Universität MünchenWZW Center of Life ScienceFreising‐WeihenstephanGermany; ^2^ Crystal Bioscience IncEmeryvilleCAUSA; ^3^ Department of Veterinary ScienceInstitute for Animal PhysiologyLudwig‐Maximilians‐Universitaet MuenchenMunichGermany

**Keywords:** Antibodies, B‐cell development, Immunoglobulins, Knockout chickens

## Abstract

Since the discovery of antibody‐producing B cells in chickens six decades ago, chickens have been a model for B‐cell development in gut‐associated lymphoid tissue species. Here we describe targeting of the immunoglobulin light chain locus by homologous recombination in chicken primordial germ cells (PGCs) and generation of VJC_L_ knockout chickens. In contrast to immunoglobulin heavy chain knockout chickens, which completely lack mature B cells, homozygous light chain knockout (IgL^−/−^) chickens have a small population of B lineage cells that develop in the bursa and migrate to the periphery. This population of B cells expresses the immunoglobulin heavy chain molecule on the cell surface. Soluble heavy‐chain‐only IgM and IgY proteins of reduced molecular weight were detectable in plasma in 4‐week‐old IgL^−/−^ chickens, and antigen‐specific IgM and IgY heavy chain proteins were produced in response to immunization. Circulating heavy‐chain‐only IgM showed a deletion of the CH1 domain of the constant region enabling the immunoglobulin heavy chain to be secreted in the absence of the light chain. Our data suggest that the heavy chain by itself is enough to support all the important steps in B‐cell development in a gut‐associated lymphoid tissue species.

## Introduction

Expression of the B‐cell receptor (BCR) on B lineage cells is crucial for B‐cell development in mammals and chickens 1, 2. The BCR consists of covalently linked immunoglobulin light and heavy chains in a noncovalent complex with the signaling molecules Igα/β. In mammals, productive VDJ_H_ rearrangement leads to expression of full‐length μ chains, which associate with the surrogate light chain to form the multimeric pre‐B‐cell receptors. Without a light chain or surrogate light chain, the heavy chain is sequestered in the endoplasmic reticulum (ER) by interactions between immunoglobulin heavy chain binding protein (BiP) and solvent‐exposed hydrophobic residues in CH1 and VH 3, 4. Signaling through the pre‐B‐cell receptor is mandatory for rearrangement of the immunoglobulin light chain in mammals 5. In contrast, VDJ_H_ and VJ_L_ rearrangement occur simultaneously in chickens 6, and therefore no pre‐B‐cell receptor or surrogate light chain is needed. Some animals, such as cartilaginous fish and camelids, have been found to express heavy chain only antibodies with no associated light chains 7. In these antibodies, the CH1 domain is absent, and the VH domains contain substitutions in the VL interface region, enabling secretion in the absence of light chain.

The original studies of B‐cell development in birds were facilitated by depletion of the B‐cell compartment using various methods, including physical ablation of the bursa 8, 9, treatment with androgens 10, irradiation 11, administration of cyclophosphamide 12, and allotype suppression 13. Genetic approaches were not possible as spontaneous mutations that affect immunoglobulin production have not been reported in poultry. Using gene targeting in chicken primordial germ cells (PGCs), cultured cells which retain the ability to transmit through the germline, we previously knocked out the immunoglobulin heavy chain 1, and now report the deletion of the light chain locus. B lineage cells in immunoglobulin heavy chain knockout (JH^−/−^) chickens lack surface immunoglobulin, cannot emigrate from the bursa of Fabricius, and are completely lacking antibodies and peripheral B cells 1. Given the role of the light chain in chaperoning the heavy chain through the ER and the agammaglobulinemic phenotype of the JH^−/−^ chickens, one would predict that the IgL^−/−^ phenotype would also be agammaglobulinemic. Here we show that although deletion of the VJC region of the immunoglobulin light chain locus eliminates IgL production, the IgL^−/−^ birds continue to produce a small amount of heavy‐chain‐only antibody from a reduced population of B cells.

## Results

### Deletion of the immunoglobulin light chain and creation of IgL^−/−^ chickens

**Figure 1:**
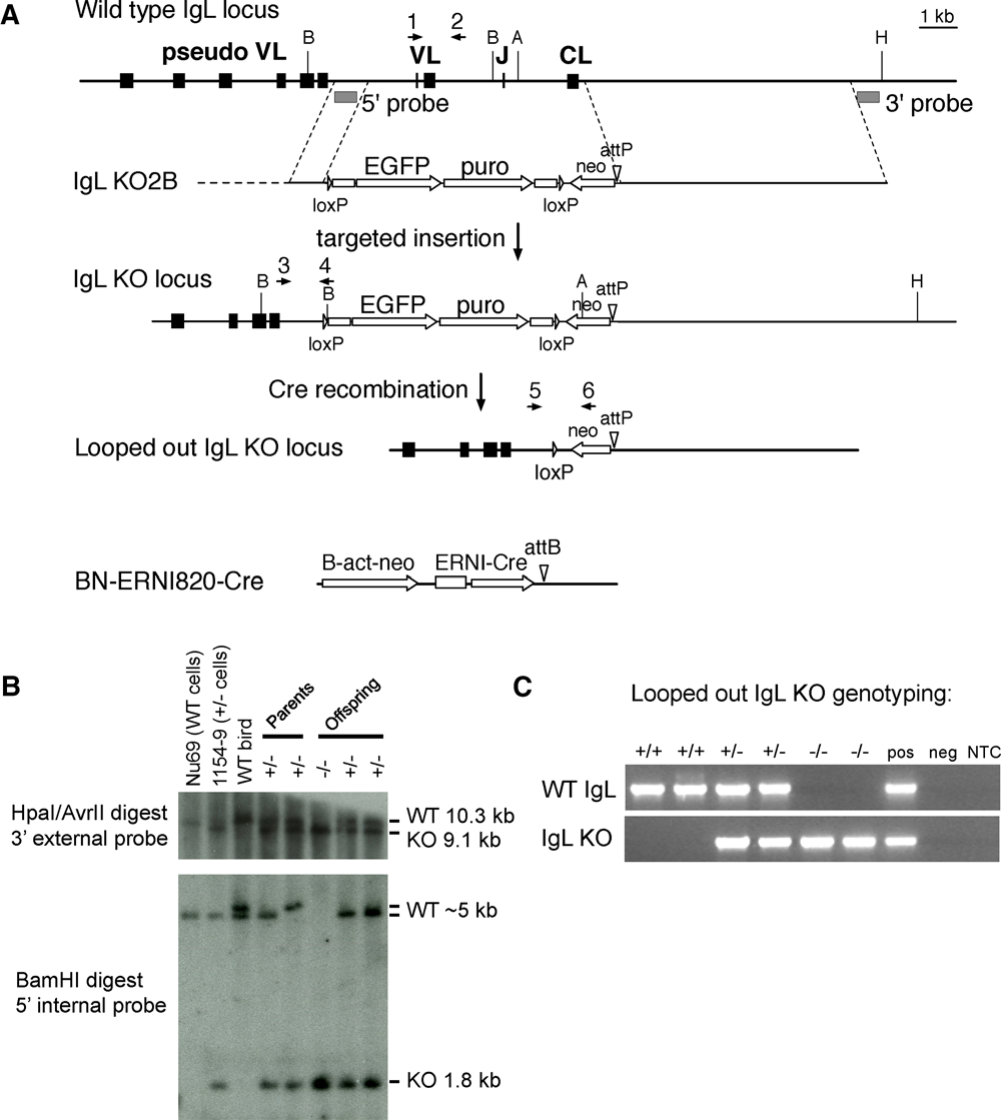
Targeting the IgL locus in chicken PGCs, removal of the selectable marker cassette, and generation of IgL^−/−^ chickens. (A) The chicken IgL locus (top line) is targeted by vector IgL KO2B (second line) with a 5′ homology region upstream of the functional V (VL) and a 3′ homology region downstream of the constant region (CL). The upstream pseudogene array (pseudo VL) is left intact in the targeted allele (third line). The positions of the 5′ and 3′ probes and restriction sites used in the Southern blot in (C) are shown. A, AvrII; B, BamHI; H, HpaI. The structure of the looped out allele obtained after Cre recombination is shown (fourth line), with the remaining promoterless neo and attP site. The ERNI‐Cre construct stably integrated into the IgL KO PGCs is shown at bottom. Scale bar, 1 kb. (B) Southern blot analysis of the original, nonlooped out IgL KO PGCs and germline progeny derived from them. The parental cell line Nu69, IgL KO clone 1154–9, and samples from wild‐type and transgenic animals are shown. Analysis of IgL^+/−^ male and female (parents) and three of their offspring are shown, with the genotypes as indicated. The light chain locus is polymorphic in our birds, and the wild‐type 5′ BamHI fragment was observed to be either 5 or 5.2 kb. (C) Genotyping looped out IgL KO birds. PCR for the wild‐type and knockout alleles was used to determine genotypes of progeny from crosses of IgL^+/−^ males and females. Two representative progeny of each genotype are shown, as indicated. Pos and neg refer to positive and negative genomic controls for the PCR reactions. NTC, no template control.

To inactivate the chicken light chain locus, we deleted all the coding exons, including the single functional V and its promoter region, the J, and the single light chain constant region (Fig. 1A). Targeting vector IgL KO2B was found to target at a frequency of 23% in PGCs (27 targeted clones out of 116 puromycin‐resistant clones screened). Correct targeting was confirmed by Southern blotting using probes internal and external to the targeting vector (Fig. 1B). An alternative vector, IgL KO2A, differing only in five single nucleotide polymorphisms found in the 5′ homology region, failed to target in 18 clones screened. A Cre recombinase construct driven by the ERNI promoter 14 was stably introduced into re‐derived knockout PGC cells to remove the β‐actin‐EGFP‐CAG‐puro selectable marker cassette (Fig. 1A), and nongreen cells were grown and injected into the vasculature of early embryos where they colonized the germline. Breeding the chimeras carrying looped out IgL KO cells generated IgL KO progeny at rates of 33–48% from four roosters (Supporting Information Fig. 1). The Cre and IgL KO transgenes segregated in the first generation (Supporting Information Fig. 1), leading to birds heterozygous for the IgL KO (IgL^+/−^) which were then intercrossed to obtain homozygous mutants (IgL^−/−^). Genotyping was performed using PCR primers specific for the wild‐type light chain and the looped out IgL KO (Fig. 1C).

Wild‐type, IgL^+/−^ and IgL^−/−^ chickens were monitored for health and gain of weight for a period of 45 days. All birds appeared to be healthy, and no significant difference in growth was observed (Supporting Information Fig. 2A). Groups of at least three birds per genotype were euthanized on embryonic day 18, day 1, and day 45 after hatch and ratios between organ and body weight were calculated. On embryonic day 18 and day 1 after hatch no significant difference was seen between IgL^+/−^ and IgL^−/−^ chickens (Supporting Information Fig. 2B and C). Forty‐five days after hatch, IgL^−/−^ birds showed significantly reduced bursa to body weight ratios compared to the control groups (Supporting Information Fig. 2D), whereas spleen to body weight ratios appeared normal.

### Low levels of peripheral B cells and plasma immunoglobulin are found in IgL^−/−^ chickens

**Figure 2:**
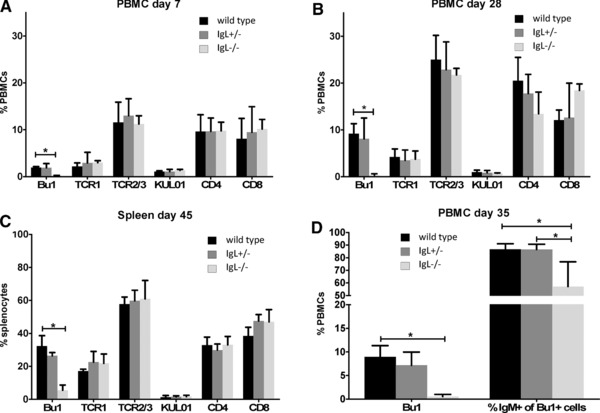
Bu1^+^ cells are detectable in blood and spleen of IgL^−/−^ chickens. (A, B) PBMCs of (A) 7‐day, (B) 28‐day‐old chickens, and (C) spleen cells of 45‐day‐old chickens were isolated and stained for B cells using Bu1‐Alexa 647, or stained for gamma/delta T cells (TCR1), alpha/beta T cells (TCR2/3), monocytes/macrophages (KUL01), and CD4^+^ and CD8^+^ cells, and detected using goat‐anti‐mouse IgG‐Alexa 647. (D) PBMCs of 35‐day‐old birds were double stained with polyclonal goat‐anti‐chIgM/donkey anti‐goat Alexa 647 and Bu1/goat‐anti‐mouse IgG‐FITC. Stained cells were analyzed by flow cytometry. The left set of bars shows the percentages of Bu1^+^ cells for the three genotypes, and the right set of bars shows the percentage of Bu1^+^ cells that are also IgM^+^. Data are shown as mean and SEM of at least four animals per genotype at each time point, following the same animals over time. Significance was calculated by Kruskal–Wallis test followed by Bonferroni correction. *p ≤ 0.05.

Seven days after hatch, up to 0.23% of peripheral blood mononuclear cells (PBMCs) in the IgL^−/−^ chickens were stained for the chicken B‐cell marker Bu1 (Bu1 is a transmembrane protein of unknown function; Fig. 2A). By 28 days, 0.34% Bu1^+^ cells were found in IgL^−/−^ chickens compared to 9.18% in wild‐type animals (Fig. 2B). In the spleen at 45 days, a population of up to 3.02% Bu1^+^ cells was found in IgL^−/−^ chickens (Fig. 2C). This was surprising since no Bu1^+^ cells were seen in chickens with a knockout of the JH segment of the immunoglobulin heavy chain 1. No significant difference between the three genotypes was seen for T cells or monocytes/macrophages at any developmental stage in PBMCs or spleen (Fig 2A–C).

Genomic DNA was isolated from PBMCs of IgL^−/−^ birds, and the VDJ region of the immunoglobulin heavy chain was amplified and sequenced for rearrangement of the locus. In 53 out of 55 sequences from IgL^−/−^ cells, in‐frame rearrangement of the immunoglobulin heavy chain VDJ was observed (Supporting Information Fig. 3). This result indicates that circulating PBMCs in IgL^−/−^ birds contain authentic B‐lineage cells, since rearrangement of immunoglobulin genes is restricted to this lineage. The high level of in‐frame rearrangement suggests that expression of the heavy chain protein is being selected during development of IgL^−/−^ B cells, since only 1/3 of the rearrangements would be in‐frame without selection. Families of sequences derived from single B‐cell clones are evident, suggesting that the heavy chain sequences are undergoing somatic hypermutation and/or gene conversion. The majority (92%) of the sequences contained noncanonical cysteines in CDR‐H3 (Supporting Information Fig. 3). A common motif was a doublet of adjacent cysteines in CDR‐H3, found in 19 of the 53 in‐frame sequences. The average CDR‐H3 length in the IgL^−/−^ sample (19 amino acids) was not significantly different from the IgL^+/−^ control (data not shown). The VH/VL interface residues were rarely mutated, despite the lack of a VL partner that would normally protect these hydrophobic amino acids from being solvent‐exposed.

To determine whether IgL^−/−^ cells express surface IgM, PBMCs from day 35 IgL^−/−^ chickens were stained with Bu1 and a polyclonal anti‐chicken‐IgM antibody. Although the percentage of Bu1^+^ cells is small (Fig. 2D, left group of bars), 57% of these cells were also IgM^+^ (Fig. 2D, right group of bars). The proportion of Bu1^+^/IgM^+^ cells is somewhat lower in IgL^−/−^ birds than in the control groups, but the staining confirms heavy chain protein expression on circulating B cells (Fig. 2D). There was no expression of the immunoglobulin light chain in bursal cells from IgL^−/−^ birds while there is expression of the immunoglobulin heavy chain (Supporting Information Fig. 4).

On days 7, 28, and 45 total immunoglobulin levels in plasma were measured by ELISA. While no IgM was detectable in IgL^−/−^ birds 1 week after hatch (Supporting Information Fig. 5a; IgY detected is of maternal origin), by 4 weeks after hatch, at which time maternal IgY is gone, low levels of IgM and IgY were measurable (Supporting Information Fig. 5B). Forty‐five days after hatch, IgM and IgY were clearly produced in IgL^−/‐^ birds, although still at levels much lower than in controls (Supporting Information Fig. 5C). Low levels of IgY were also found in egg yolks from IgL^−/−^ laying hens (Supporting Information Fig. 5D).

### IgL^−/−^ B cells show a deletion of the immunoglobulin heavy chain CH1 domain

**Figure 3:**
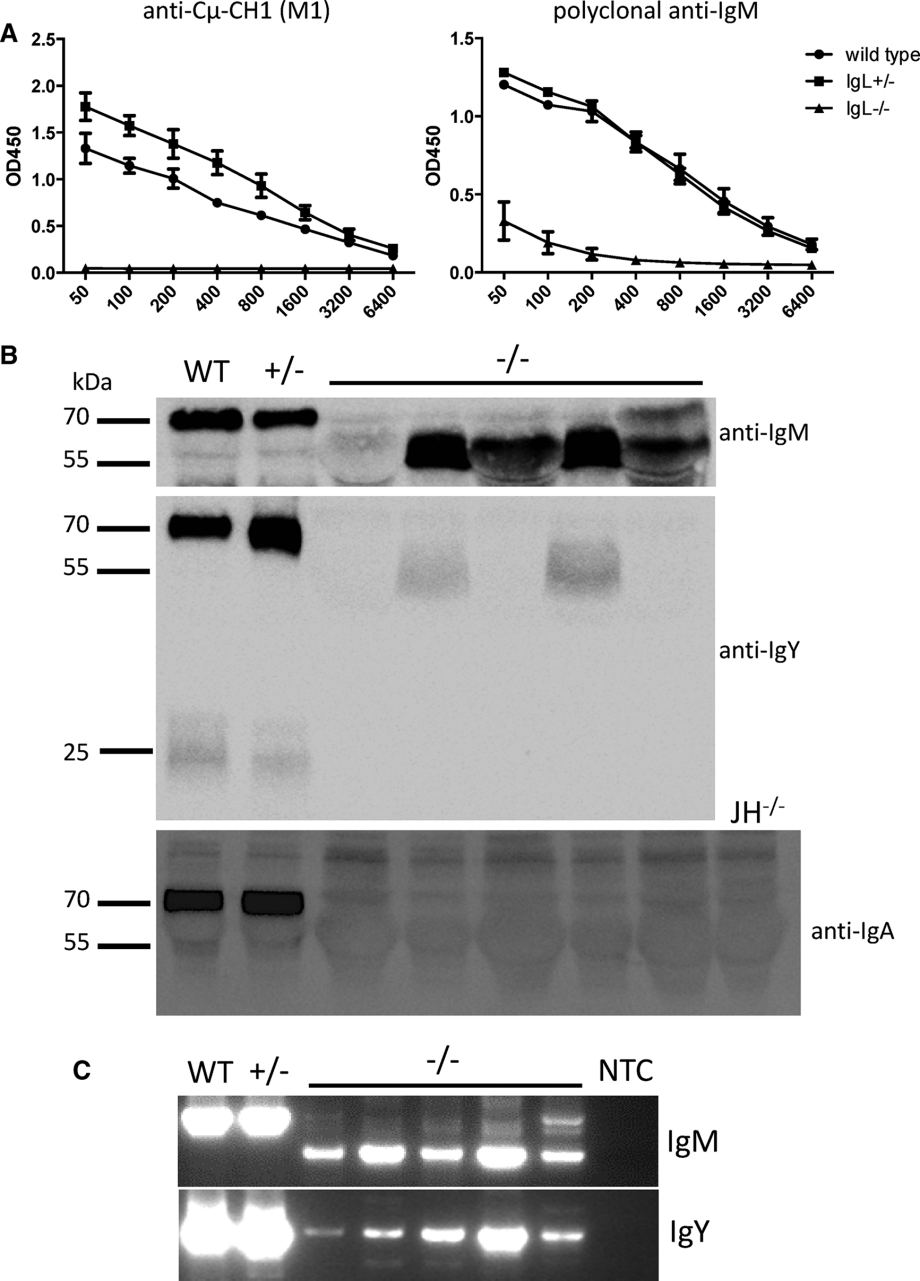
The IgM and IgY heavy chain CH1 domain is deleted in IgL^−/‐^ chickens. (A) ELISA plates were coated with anti‐chicken‐Cμ‐CH1 (M1) which binds the CH1 domain. Plasma of 28‐day‐old birds was incubated on the plates and captured IgM was detected with polyclonal goat‐anti‐chicken‐IgM‐POD. For each graph, data are shown as mean and SD of at least four birds per genotype. The IgL^−/−^ ELISA from Supporting Information Fig. 5B, using a polyclonal anti‐IgM as capture antibody, is reproduced here for comparison. (B) Plasma samples of one wild‐type (WT), one IgL^+/−^, and five IgL^−/−^ birds were tested by Western blot for IgM, IgY, and IgA. Serum from a heavy chain knockout (JH^−/−^) bird is included as a control for background. (C) RNA was isolated from PBMCs of the same 28‐day‐old chickens used in (B) and one‐Step‐RT‐PCR was performed spanning the region from the heavy chain V to the CH2 domain of IgM or IgY. NTC, no template control. (B, C) PCR data shown are from single experiments representative of eight performed.

Since the CH1 domain of the immunoglobulin constant region normally associates with either BiP in the ER which leads to retention of the protein, or a light chain constant region leading to secretion, we considered the possibility that a deletion of CH1 in the immunoglobulin heavy chain enables its secretion without an associated light chain. To test whether the CH1 domain is missing, ELISA plates were coated with a monoclonal antibody against the CH1 domain (Cμ‐CH1) and plasma of 28‐day‐old IgL^−/−^ chickens was incubated on the plates. Captured IgM was detected with a polyclonal anti‐chicken‐IgM antibody. No signal was detected in IgL^−/−^ birds, while the control groups showed high titers of IgM (Fig. 3A; the day 28 IgM ELISA from Supporting Information Fig. 5B, which used a polyclonal anti‐IgM as the capture antibody, is reproduced here for comparison). Western blot analysis of plasma from 45‐day‐old birds with a polyclonal anti‐chicken‐IgM antibody revealed a band at approximately 56 kDa for IgM in IgL^−/−^ chickens, compared to 72 kDa in controls. One IgL^−/−^ bird also showed a faint band at approximately 72 kDa. The same difference in molecular weight was seen for IgY (IgL^−/−^ birds ∼55 kDa, control birds 70 kDa) (Fig. 3B). A band at 25 kDa representing the immunoglobulin light chain was visible in wild‐type and IgL^+/−^ chickens, but absent in IgL^−/−^ chickens (Fig. 3B). Serum IgA was seen in wild‐type and IgL^+/−^ birds, but was not detected above background in the IgL^−/−^ birds (Fig. 3B).

RNA was isolated from PBMC of wild‐type, IgL^+/−^ and IgL^−/−^ chickens and RT‐PCR was performed to amplify IgM using a reverse primer located in the CH2 domain of the constant region and a forward primer binding in the leader region of the VDJ. Wild‐type and IgL^+/−^ showed a band at ∼800 bp while IgL^−/−^ chickens showed a band at ∼500 bp, matching a deletion of CH1 (Fig. 3C). Faint bands of the same size as those seen in wild‐type and IgL^+/−^ chickens (∼800 bp) were also seen in IgL^−/‐^ birds, but their provenance at the present time is unclear. Sequencing of the lower bands (∼500 bp) revealed a deletion of CH1 in the IgL^−/−^ chickens (Supporting Information Fig. 6). RT‐PCR for IgY (Fig. 3C) showed predominantly full‐length products, which were confirmed by cloning and sequencing. Lower bands observed for IgY were determined to be nonspecific amplicons.

### Immunoglobulin light chain knockout chickens can still produce antigen‐specific antibodies

**Figure 4:**
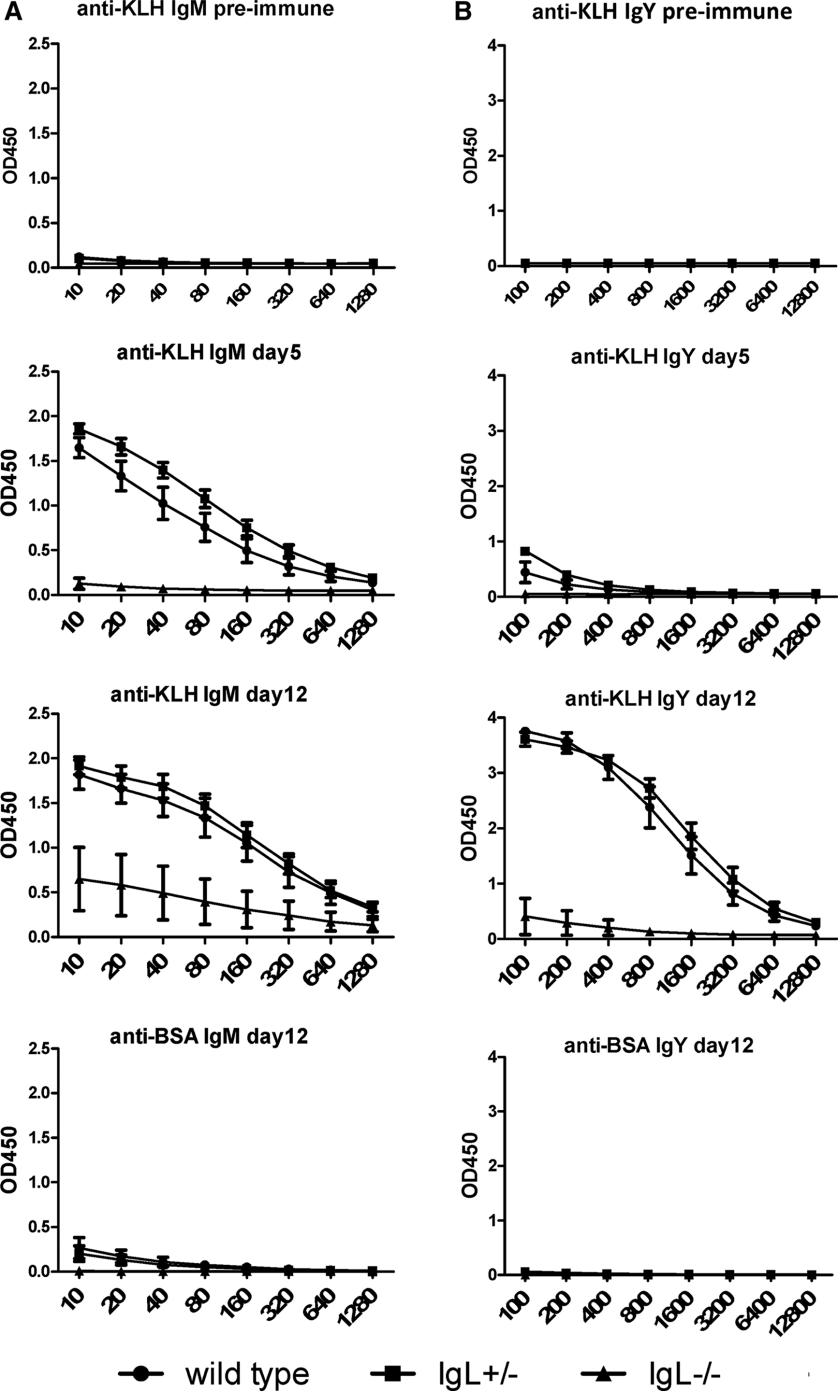
IgL^−/−^ chickens produce antigen specific immunoglobulin. (A, B) 35‐day‐old chickens were immunized with keyhole limpet hemocyanin (KLH) and boosted 7 days after the initial immunization. Levels of antigen‐specific (A) IgM and (B) IgY were analyzed 5 days and 12 days after immunization. Data are shown as mean and SEM of at least four birds per genotype, following the same animals over time.

In order to check if IgL^−/−^ chickens are capable of producing antigen‐specific, heavy‐chain only antibodies, groups of 4‐week‐old chickens were immunized with keyhole limpet hemocyanin (KLH). Plasma samples were analyzed for the presence of antigen‐specific IgM (Fig. 4A) and IgY (Fig. 4B) by ELISA over time. No titer against KLH was detectable in any group prior to immunization. By five days after immunization, a small amount of antigen‐specific IgM was present in the IgL^−/−^ chickens. Although their responses were significantly lower than wild‐type and IgL^+/−^ birds, four out of five IgL^−/−^ chickens showed antigen‐specific IgM and three out of five produced KLH‐specific IgY by day 12 (Fig. 4B). No titer against an unrelated protein (BSA) was observed in the KLH‐immunized birds, indicating that the immune responses were specific to KLH.

### B‐cell follicles can develop cortical structures of reduced size in the bursa of IgL^−/−^ chickens

**Figure 5:**
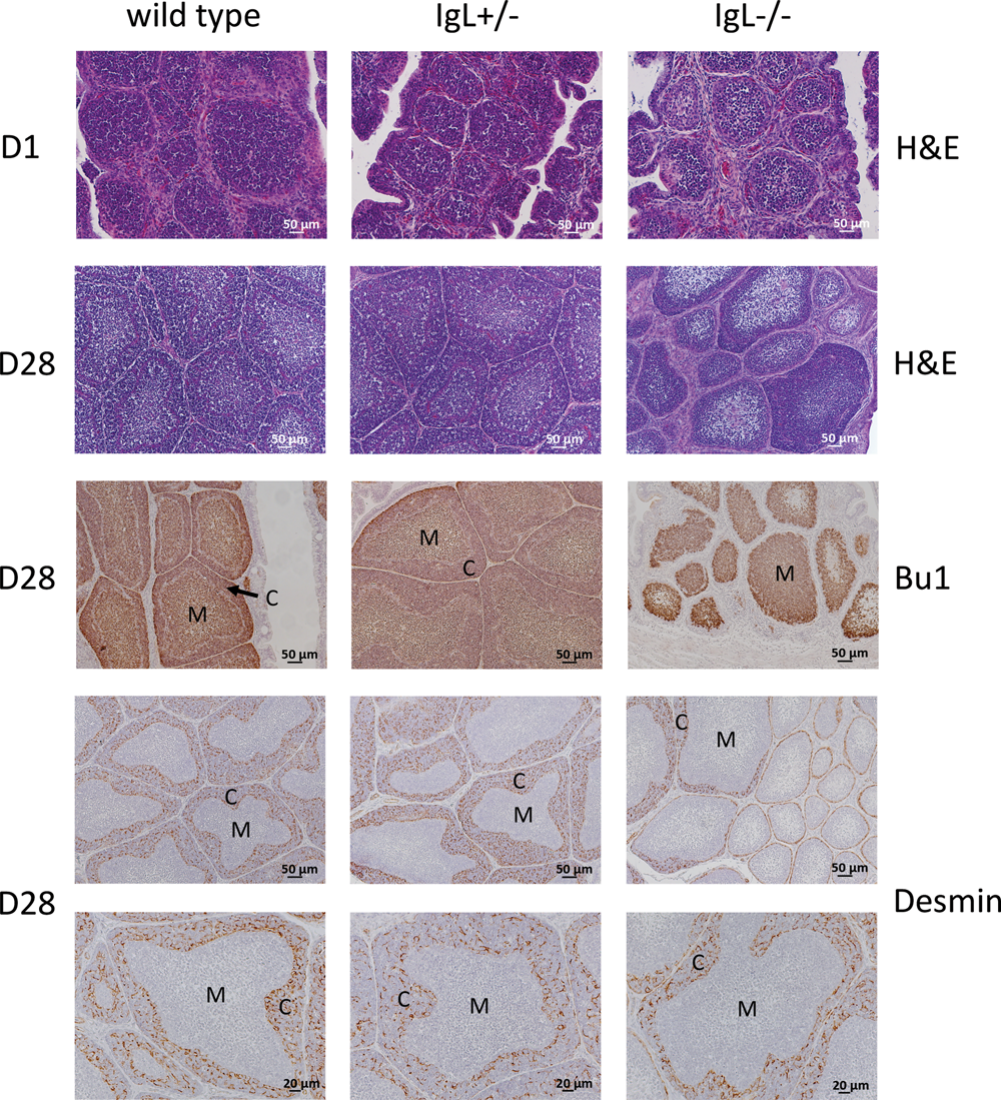
Formation of B‐cell follicles in the bursa of IgL^−/−^ chickens. Sections of paraffin‐embedded bursa tissue from 1‐day and 28‐day‐old wild‐type, IgL^+/−^ and IgL^−/−^ chickens were stained with hematoxylin and eosin (H&E). Sections from day 28 were also stained with a chicken B‐cell marker (anti‐chicken Bu1a and Bu1b) and the basement membrane forming the cortico‐medullary junction was stained using a cross‐reactive anti‐human‐desmin antibody. Cortex (C) and medulla (M) are indicated. Antibodies were detected using the Vector ABC Kit followed by the Vector DAB Kit. One representative picture per group and staining is shown. Three bursas per genotype with a minimum of ten sections per organ were analyzed.

Bursas of 1‐day, 28‐day, and 45‐day‐old chickens were fixed and embedded for sectioning. Hematoxylin and eosin (H&E) staining of bursa sections on day 1 did not show a difference between the genotypes (Fig. 5). In contrast, 4 weeks after hatch wild‐type and IgL^+/−^ animals showed well‐developed B‐cell follicles with separation into medulla and cortex, whereas most follicles in the IgL^−/−^ birds were small and depleted of lymphocytes and showed little cortical development. Some follicles, however, were found to contain lymphocytes and showed a separation into medulla and cortex. All follicles were positive for the B‐cell marker Bu1. The basement membrane forming the cortico‐medullary junction was visualized using a cross‐reactive anti‐human‐desmin antibody. B cells in some follicles of IgL^−/−^ chickens migrated across the basement membrane to form medulla and cortex (Fig. 5).

### B cells of IgL^−/−^ chickens express heavy chain only IgM

**Figure 6:**
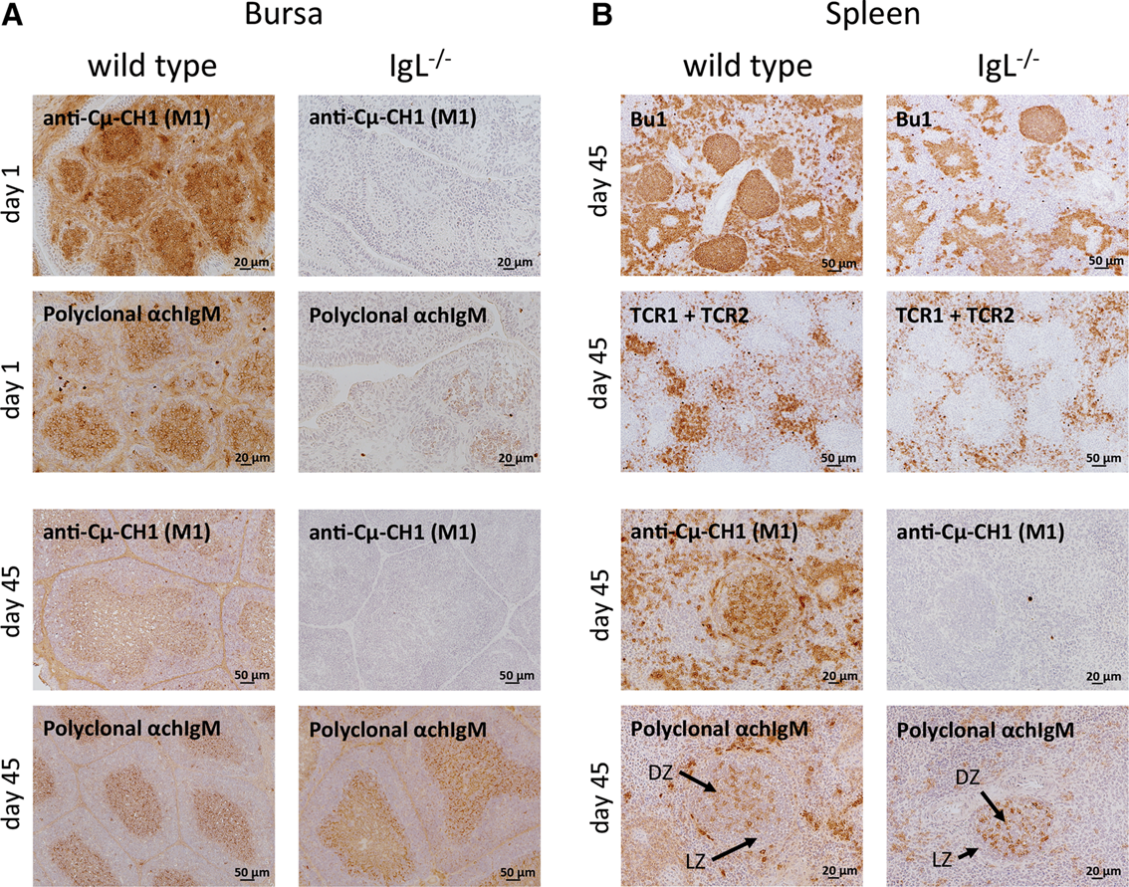
A single chain immunoglobulin heavy chain supports B‐cell development in IgL^−/−^ chickens. (A, B) Fresh frozen sections of (A) bursa and (B) spleen from 1‐day and 45‐day‐old wild‐type and IgL^−/−^ chickens were stained for B cells (anti‐chicken‐Bu1 (AV20)), T cells (TCR1 + TCR2), anti‐Cμ‐CH1 domain (M1) and IgM (polyclonal anti‐chIgM). Antibodies were detected using goat‐anti‐mouse‐HRP followed by the Vector DAB Kit. One representative picture per group and staining is shown. Three spleens and bursas per genotype with a minimum of ten sections per organ were analyzed.

Sections of the bursa (Fig. 6A) of wild‐type and IgL^−/−^ birds were analyzed for expression of IgM using the monoclonal anti‐Cμ‐CH1 (M1) or polyclonal anti‐IgM. No staining was seen on day 1 using anti‐Cμ‐CH1 in IgL^−/−^ chickens. In contrast the polyclonal anti‐IgM at day 1 showed weak cell‐surface expression, although not all follicles were positive for IgM. On day 45 after hatch, staining with the polyclonal anti‐IgM in the well‐developed bursal follicles in the IgL^−/−^ chickens was equivalent to that seen in the wild‐type and IgL^+/−^ control birds (Fig. 6A).

### IgL^−/−^ chickens form germinal centers upon immunization

Sections of the spleen of wild‐type and IgL^−/−^ birds at day 45 were stained for B cells (Bu1) and T cells (TCR1/2). Although the density of germinal centers was lower in the IgL^−/−^ animals, the germinal center structure appeared the same as in wild‐type (Fig. 6B). No difference in B‐ and T‐cell staining was detected between the two groups, and B‐cell and T‐cell areas showed the same properties. Germinal centers with a dark and light zone could be seen in both genotypes. While staining with the monoclonal anti‐Cμ‐CH1 was negative in IgL^−/−^ birds, their B cells stained with the polyclonal anti‐IgM (Fig. 6B).

### Peripheral B cells in IgL^−/−^ chickens emigrate from the bursa

To determine if the Bu1^+^ cells seen in IgL^−/−^ chickens originated from the bursa, labeling of bursal B cells was performed by applying FITC solution to the anal lips of 6‐day‐old birds. Ten hours after application, circulating B cells were analyzed for co‐labeling with FITC. In wild‐type, IgL^+/−^ and IgL^−/−^ chickens, FITC^+^/Bu1^+^ cells were detectable (Supporting Information Fig. 7A–C, respectively). Although very few Bu1^+^ cells in the IgL^−/−^ birds were FITC^+^, the presence of double‐labeled cells in circulation suggests these Bu1^+^ cells originated in the bursa.

## Discussion

Throughout the history of domestication, thousands of spontaneous mutations have been incorporated into the genome of hundreds of breeds of chickens. By contrast, application of the tools of cell and molecular biology to create and integrate specific mutations into the genome is a recent activity. Several lines expressing randomly integrated GFP have been reported 15, 16, 17, 18, and we previously described the phenotype of birds in which the JH region of the immunoglobulin heavy chain was deleted by homologous recombination in PGCs 1. This report extends the range of technologies for engineering the chicken genome by knocking out the immunoglobulin light chain, as well as excising transgenes flanked by loxP sites using Cre recombinase, in PGCs. Recombinases are used in many different ways in both cell culture and transgenic mice 19 and the proof of concept shown here creates new opportunities for similar applications in engineering the genome of birds.

The phenotype of JH^−/−^ chickens is characterized by a lack of peripheral B cells, absence of the IgH protein and a corresponding absence of antibody 1. Although a similar phenotype was anticipated in the IgL^−/−^ birds, the outcome corresponds only in part. As predicted, IgL^−/−^ birds do not produce an IgL protein and many, but not all bursal follicles, fail to develop. Anatomically, the nonatretic follicles form a typical medulla and cortex structure. The presence of some apparently normal bursal follicles is associated with an unusual population of B cells producing heavy‐chain‐only IgM antibodies that lack the CH1 domain of the constant region (ΔCH1). The mixed population of atretic and apparently normal bursal follicles suggests that the follicles are supported only if the B cell or cells seeding the bursal follicle quickly gain the capacity to generate a ΔCH1 constant region. The active bursal follicles appear to correctly process the ΔCH1 heavy‐chain only antibodies because bursal cells can migrate into the periphery, where their V regions are found to be correctly rearranged and in‐frame. Furthermore, the B‐cell population responds by forming antigen‐specific, ΔCH1 heavy‐chain only IgM and truncated IgY antibodies. Although unexpectedly we could not detect an IgY CH1 deletion by RT‐PCR, it is possible that a small amount of mRNA, below detection, is spliced to remove the CH1 domain, which results in the small amount of truncated plasma IgY. Taken together, these data support the conclusion that the truncated heavy‐chain only immunoglobulin molecule is sufficient to support B‐cell differentiation from genesis of the lineage to antibody‐producing plasmablasts.

Naturally occurring heavy‐chain only antibodies have been found in sharks 20 and camelids 7. In camels, single chain heavy chain antibodies show longer CDR3 regions compared to regular heavy chain molecules 21, 22, 23. Analysis of the heavy chain CDR3 regions of IgL^−/−^ chickens did not show differences in length compared to heavy chain CDR3 regions of wild‐type birds. Heavy chain‐antibodies from camels and sharks, and H+L antibodies from wild‐type chickens, often contain noncanonical cysteines in CDR3, which may form disulfide bonds with noncanonical cysteines elsewhere in the variable region 24, 25, 26. Paired cysteines within CDR3 are often observed that could form intra‐CDR disulfide bonds to stabilize loop secondary structures. Although we observed several examples of possible noncanonical cysteine pairs in the heavy chain‐only antibodies from IgL^−/−^ birds, a more prevalent pattern was two adjacent cysteines in CDR3, with no additional cysteines in the variable region with which to pair. This pattern is not unique to the heavy‐chain only antibodies in IgL^−/−^ chickens, as we have also observed it in wild‐type birds, but it has not been reported by others 26. Adjacent cysteines are encoded by at least two germline D segments in chickens 27, a potential source for this pattern. The VH domains did not contain substitutions of the hydrophobic residues found at the VL/VH interface in these bulk samples, although we did not analyze the sequence of antigen‐specific antibodies to see if such mutations were selected in the immune response.

Mice lacking Ig kappa and Ig lambda 28, 29 also produce a ΔCH1 heavy chain antibody but there are some notable differences in the IgL^−/−^ phenotype. In chickens, B‐cell development is fully supported at least in some bursal follicles and the spleen appears to be functioning normally. In contrast, development stops at the pre‐B2 to immature B‐cell stage in IgL^−/−^ mice 30. Furthermore, B‐cell development in the bone marrow is fully retained but secondary lymphoid organs are completely depleted of B cells. No IgM is found in IgL^−/−^ mice 30.

In mammals three different hypotheses are given for the deletion of CH1: alternative splicing, splice‐site mutation or exon deletion. All three events have been seen in human and mouse B cells 31, 32. Since we observed deletions in IgM and IgY at the protein level, we assume that the underlying mechanism for the deletion of CH1 is related to an alternative splicing process and not to a deletion caused by faulty class‐switching at the genomic DNA level. However, we cannot exclude a combination of faulty class‐switching and alternative splicing. Comparison of VDJC sequences from IgL^−/−^ and wild‐type did not reveal an obvious motif driving error‐prone splicing that would delete the CH1 domain, although we did not sequence genomic CH1 exons where such mutations may also occur.

In conclusion, the phenotype of IgL^−/−^ chickens is different from that of IgH^−/‐^ chickens and light‐chain deficient mice. The unusual ΔCH1 heavy‐chain only antibodies and their association with bursal follicle development and B‐cell emigration from bursal follicles into the periphery provides new information about the ontogeny of the immune system of birds and the plasticity of B‐cell signaling through the B‐cell receptor.

The inactivated immunoglobulin locus can be reconstructed with components encoding human V regions 33, 34 to produce chickens with human‐chicken chimeric immunoglobulin light chains. In combination with a similarly reconstructed heavy chain, we can design chickens from which antibodies with fully human antibody binding domains can be recovered.

## Materials and methods

### Animal experiments

Commercial Brown and White Leghorn chickens and Minnesota Marker Line chickens were used. Animal experiments were done in accordance to IACUC approved protocols and under supervision of the IACUC committee.

### Targeting vector construction

Homology regions of 1023 bp and 7196 bp were amplified from the PGC line used for targeting experiments, Nu69 (Fig. 1). The 5′ homology region lies 1290 bp upstream of the IgL ATG start site, and the 3′ homology region begins 190 bp downstream of the stop codon. Vectors IgL KO2A and 2B contain polymorphic 5′ homology regions from the two different alleles present in Nu69. The selectable marker cassette was identical to that in Schusser *et al*. 1.

### ERNI‐Cre construct

The chicken ERNI promoter was amplified using primers ERNI fw1 5′‐TGATTTGGGGGATCCGGGGTGAAGGGTGGATGTTTATTAG‐3′ and ERNI rev1 5′‐AGAACTAGTGGATCCGAATTCTAGTTGGCAGAGAACCCCTCAAGTC‐3′ with overhangs for cloning. The ERNI promoter was cloned upstream of the Cre‐myc gene (Connie Cepko, Harvard). A β‐actin‐neo selection cassette and an attB site were added to the construct for stable integration using phiC31 integrase.

### Transfection

PGCs were cultured and transfected as previously described 18. Transfected clones were screened for targeted integration by PCR with a primer in the IgL locus upstream of the location of the 5′ homology region (Primer 3 in Fig. 1: L‐40303‐F, 5′‐ACTGTGCTGCAGGTGGCTATG‐3′) and a reverse primer in the vector just downstream of the 5′ loxP site (Primer 4 in Fig. 1: HA‐R, 5′‐ATACGATGTTCCAGATTACGCTT‐3′). Correct structure of the targeting event was confirmed by Southern blot using probes derived from the light chain locus (Fig. 1). Southern blotting was performed as described in Schusser *et al*. 1.

### Chimera production and germline testing

The original IgL KO clones 1153–8 and 1154–9 were injected into recipient embryos and male chimeras were raised to sexual maturity. Chimeras were bred to wild‐type females and germline progeny at embryonic stages were used to re‐derive a new knockout PGC line, 229–92 35. These cells were stably transfected with the ERNI‐Cre construct. Four clones showed loopout of the selectable markers, as judged by loss of green fluorescence and by PCR. These clones were injected to produce chimeras, which were bred to obtain looped out IgL knockout birds without the Cre transgene, which segregated independently.

### Genotyping

Genomic DNA was obtained from comb biopsies on the day of hatch or at embryonic day 18. The wild‐type allele was detected with primers 1 and 2 in Fig. 1 (cVL‐L‐F: 5′‐ATGGCCTGGGCTCCTCTCCTC‐3′ and L‐43365‐R: 5′‐GAGCAGATGAGGAGCTGCCAGC‐3′), product size 709 bp, and the looped out knockout allele with primers 5 and 6 in Fig. 1 (L‐41282‐F: 5′‐AGATCTCCTCCTCCCATCCTG‐3′ and neo‐F2: 5′‐GGTTCGAAATGACCGACCAAGC‐3′), product size 618 bp. The Cre gene was detected with primers Cre‐seqF2 (5′‐TATGCGGCGGATCCGAAAAG‐3′) and Cre‐R2 (5′‐CTTCCAGGGCGCGAGTTGAT‐3′), product size 440 bp.

### Flow cytometry

PBMCs were isolated by Ficoll (Sigma Aldrich) density gradient centrifugation from EDTA‐treated blood or splenocyte cell suspensions. The following mouse‐anti‐chicken antibodies were used: Bu‐1 (AV20, conjugated or unconjugated), lambda‐PE (L‐1), TCR gamma/delta (TCR1), TCRαβ/Vβ1 (TCR2), TCR αβ/Vβ2 (TCR3), monocytes/macrophage marker (KUL01), Cμ‐CH1 (M1, conjugated or unconjugated), CD4 (CT4), and CD8 (CT8) (all from Southern Biotech), ms‐anti‐chicken‐Cμ (BK) made by Bernd Kaspers (binds a part of the constant region of Cμ other than CH1), or goat‐anti‐chicken‐IgM (Bethyl Laboratories). Secondary antibodies were: goat‐anti‐mouse‐IgG‐Alexa‐647 (Rockland), goat‐anti‐mouse‐IgG‐FITC (Thermo Fisher Scientific), or donkey‐anti‐goat‐IgG‐Alexa‐647 (Rockland). Fluorescence was measured using an Attune^TM^ Flow Cytometer (Thermo Fisher Scientific).

### ELISA

To measure total plasma IgM and IgY EIA plates (Corning) were coated with 2 μg/mL goat‐anti‐chicken‐IgM (Bethyl Laboratories) or 0.8 μg/mL goat‐anti‐chicken‐IgY (Sigma Aldrich) overnight at 4°C. Plates were blocked with PBS/3% skim milk and incubated with chicken plasma. Bound IgM or IgY was detected with goat‐anti‐chicken‐IgM‐HRP (Bethyl Laboratories) or goat‐anti‐chicken‐IgY‐HRP (Sigma Aldrich) and developed with TMB substrate (Thermo Fisher Scientific). OD was measured at 450 nm. To measure antigen‐specific IgM and IgY, plates were coated with 10 μg/mL KLH or BSA. For detection of the Cμ‐CH1 domain plates were coated with 2 μg/mL ms‐anti‐chicken‐ Cμ‐CH1 (M1) (Southern Biotech). All subsequent steps were performed as for detection of total plasma IgM and IgY.

### Immunization

Four‐week‐old birds were immunized by intramuscular injection using 300 μg keyhole limpet hemocyanin as described 1.

### RT‐PCR

Total RNA was extracted from PBMC using the RNeasy kit (Qiagen) and subjected to PCR with reverse transcription using the OneStep kit (Qiagen) or Reverse Transcriptase System (Promega). IgM and IgY transcripts were amplified with primers chVH‐F9 (5′‐CACCAGTCGGCTCCGCAACCATG‐3′), cIgM‐CH2‐R (5′‐ GGGGTGCATGGTGACGAAAAG‐3′), and cIgY‐CH3‐R (5′‐GCACCTCAGTTTGGCGTCTA‐3′**)**.

### PCR and sequencing of IgH

The rearranged chicken heavy chain was amplified from genomic DNA using a primer upstream of the VH exon (chVH‐F9: 5′‐CACCAGTCGGCTCCGCAACCATG‐3′) and a primer downstream of the JH exon (chJC‐R45: 5′‐GCCCAAAATGGCCCCAAAAC‐3′). Products (∼1 kb) were TA cloned (Thermo Fisher Scientific) and sequenced using a primer in the VH framework region 1.

### Western blot

Immunoglobulins were detected using: goat‐anti‐chicken‐IgM‐HRP (Bethyl), goat‐anti‐chicken‐IgY‐HRP (Jackson ImmunoResearch Laboratories), and goat‐anti‐chicken IgA (Bethyl). Blots were developed with ECL substrate (GE Healthcare) and signal detected using the MicroChemi System (DNR Bio‐Imaging Systems).

### In vivo labeling

One hundred microliters of a 5 mg/mL FITC solution (Sigma) were applied to the anal lips of chickens 6 days after hatch and taken up by anal sucking movements as described elsewhere 36, 37. FITC labeled B cells were analyzed 10 h after application.

### Histology

Sections were prepared as described before 1. The following antibodies were used: ms‐anti‐desmin (D33) (Thermo Scientific), the mouse‐anti chicken antibodies Cμ‐CH1 (M1), Bu‐1a, Bu1b, TCRgamma/delta (TCR1), TCRab/Vb1 (TCR2) (all Southern Biotech), and goat‐anti‐chicken‐IgM‐HRP (Bethyl Laboratories). Antibodies were detected using the Vector ABC Kit (paraffin sections) or goat‐anti‐mouse‐HRP (Jackson ImmunoResearch Europe) for 30 min (cryosections) followed by the Vector DAB Kit (Vector). Sections were counterstained with Mayer's hematoxyline (Medite). Eukitt mounting medium (EMS) was used to mount the stained sections. Pictures were taken using a Zeiss Axioskop equipped with an AxioCam MRc5 and the AxioVision software.

### Statistics

Mean and standard error of the mean (SEM) are shown. Normally distributed data (Shapiro–Wilk tests) were analyzed by one‐way ANOVA followed by Bonferroni correction of α. Non‐normally distributed data (Shapiro–Wilk tests) were analyzed by nonparametric test (Kruskal–Wallis tests) followed by Mann–Whitney‐U‐test with Bonferroni correction of α. Differences were considered statistically significant if *p* was ≤ 0.05. Data were analyzed using SPSS version 22 (IBM).

## Conflict of interest

Crystal Bioscience is commercializing a human antibody‐expressing chicken incorporating the light chain knockout described.

**Abbreviation**
PGCs: primordial germ cells

## Supporting information

As a service to our authors and readers, this journal provides supporting information supplied by the authors. Such materials are peer reviewed and may be re‐organized for online delivery, but are not copy‐edited or typeset. Technical support issues arising from supporting information (other than missing files) should be addressed to the authors.

Supporting Information

Peer Review Correspondence
