# Supplementary material for: Expression of heavy chain‐only antibodies can support B‐cell development in light chain knockout chickens
Source: Eur J Immunol. 2016 Aug 2;46(9):2137–48. doi: 10.1002/eji.201546171 (PMC5113765; doi:10.1002/eji.201546171)
Supplement: Supplementary file 1 — Supporting Information [file EJI-46-2137-s001.pdf]

European Journal of Immunology

Supporting Information
for
DOI 10.1002/eji.201546171

Benjamin Schusser, Ellen J. Collarini, Darlene Pedersen, Henry Yi, Kathryn Ching, Shelley Izquierdo, Theresa Thoma, Sarah Lettmann, Bernd Kaspers, Robert J. Etches, Marie-Cecile van de Lavoir, William Harriman and Philip A. Leighton

Expression of heavy chain-only antibodies can support B-cell development in light chain knockout chickens

© 2016 The Authors. *European Journal of Immunology* published by WILEY-VCH Verlag GmbH & Co. KGaA, Weinheim.

A

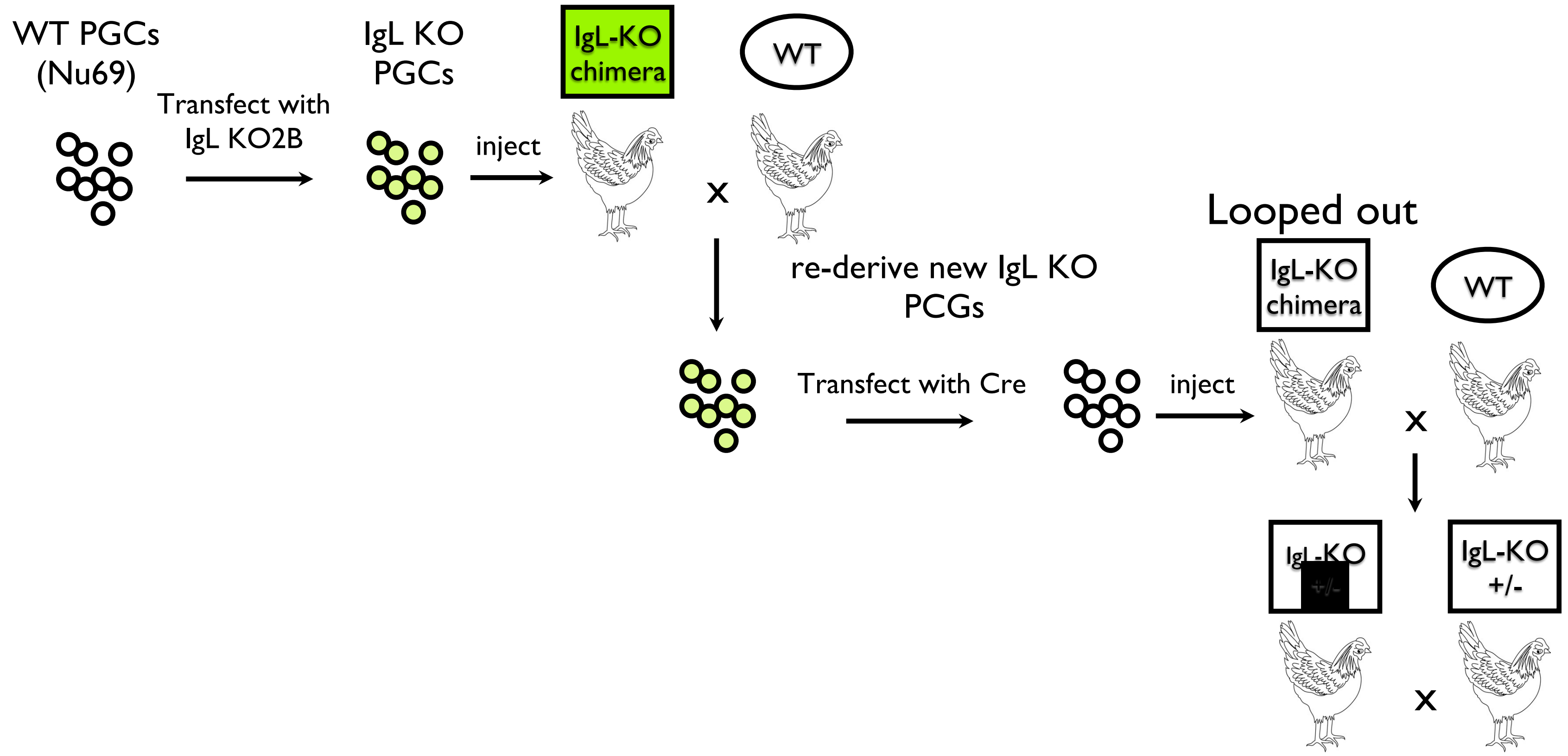

B

Germline transmission rates of original and looped out IgL knockout cell lines

Cell line	Construct	# roosters	Total progeny	IgL KO progeny	% IgL KO progeny
1153-8	IgL KO2B	6	2180	12	0.6
1154-9	IgL KO2B	4	1378	450	33
1565-1A	IgL KO/Cre	1	24	8	33
1574-1	IgL KO/Cre	5	509	202	40
1574-2	IgL KO/Cre	2	87	41	47
1574-4	IgL KO/Cre	2	151	73	48

Supporting Information 1. Breeding strategy to obtain $IgL^{-/-}$ chickens without the selectable marker cassette.
A. To obtain chickens with a knockout of the immunoglobulin light chain, PGCs were transfected with targeting construct IgL KO2B, replacing the VJC of the immunoglobulin light chain with a selectable marker cassette containing EGFP. Chimeras generated by the resulting PGC line were bred to wild type birds, resulting in embryonic offspring from which PGC cell lines with a knockout of the immunoglobulin light chain were re-derived. $EGFP^{+}$ $IgL^{+/-}$ cells were stably transfected with a construct coding for Cre recombinase to loop out the selectable marker cassette. Resulting $EGFP^{-}$ PGCs were used to generate $IgL^{+/-}$ chickens without a selectable marker cassette. The Cre and IgL KO transgenes were unlinked and birds containing only the IgL KO were kept for further breeding and analysis.
B. Germline transmission rates of the two original, non-looped out IgL KO PGC lines (upper two rows) and the four Cre/looped out IgL KO cell lines (lower four rows).

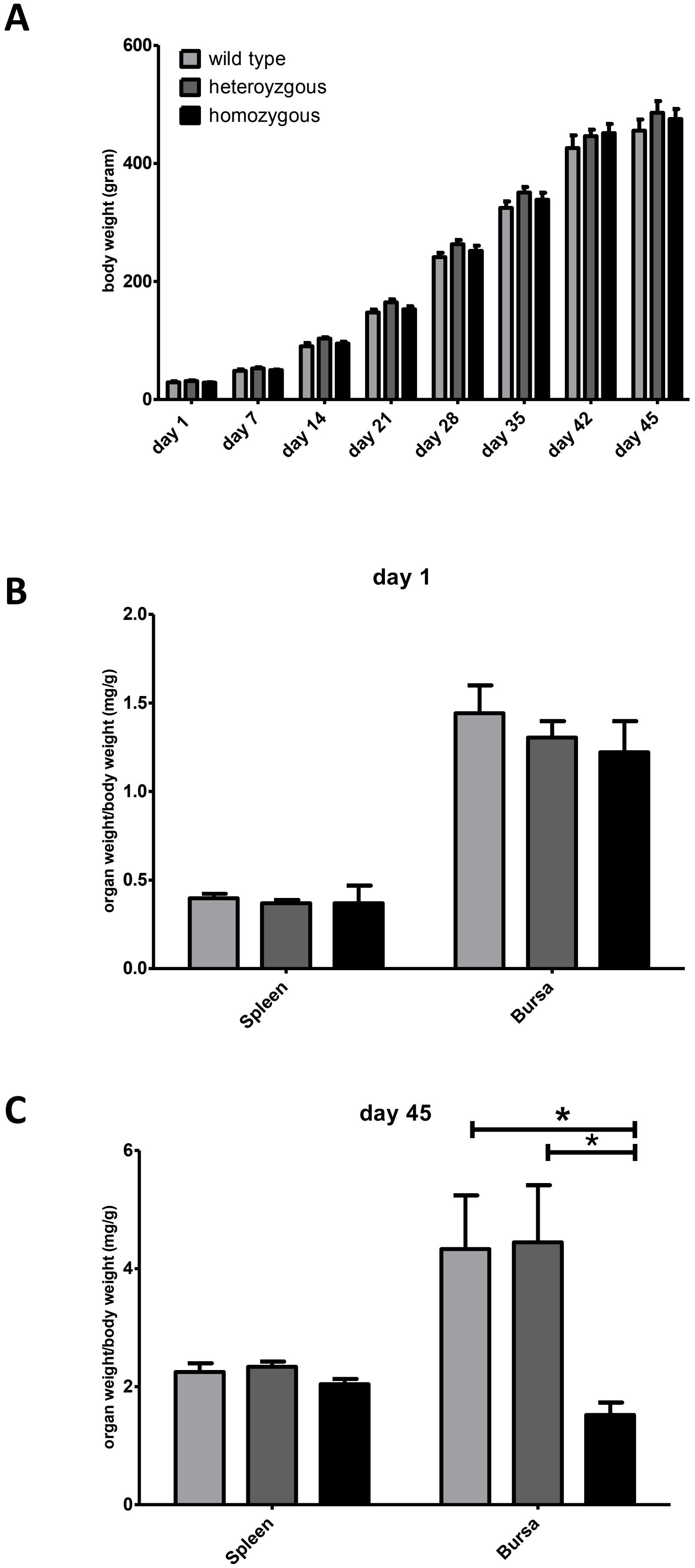

Supporting Information 2. IgL$^{-/-}$ chickens show similar body weights as wild type but have decreased bursa weights.

The body weights (A) of wild type, IgL$^{+/-}$ and IgL$^{-/-}$ chickens were monitored for 45 days after hatch. The weights of spleens and bursas on day 1 (B) and day 45 (C) after hatch were taken. The ratio between organ and body weight is shown. Mean and SEM of at least four birds per group and time point are shown. Significance for day 45 was calculated by ANOVA followed by Bonferroni correction. * $p \leq 0.05$.

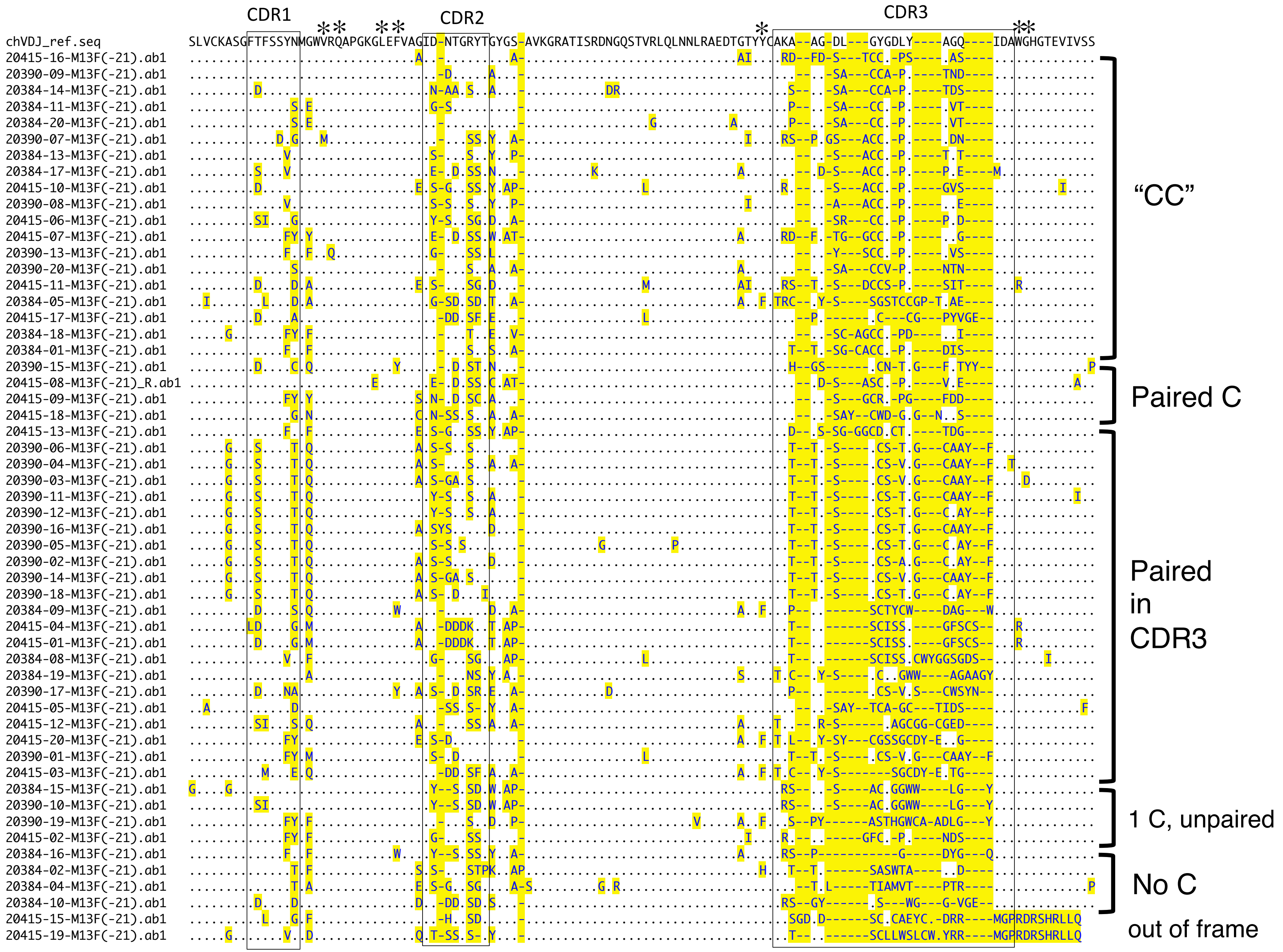

Supporting Information 3. IgL$^{-/-}$ chickens show in frame VDJ rearrangement of the immunoglobulin heavy chain.
Genomic DNA was isolated from PBMCs of 28 day old IgL$^{-/-}$ chickens. Rearranged heavy chain VDJ was amplified using oligos spanning the VDJ region and cloned into pCR2.1. Out of 60 clones sequenced, 55 unique sequences were recovered. The unique sequences are shown aligned to a theoretical reference sequence. 53 out of the 55 sequences were in-frame. The sequences were grouped according to noncanonical cysteine content, as indicated to the right. The designations referring to noncanonical cysteines are: "CC", two adjacent cysteines present in CDR3 (a few of these sequences contained a third cysteine in CDR3); Paired C, two cysteines in different regions of the V that could potentially form an intrachain disulfide bond with each other; Paired in CDR3, two cysteines within CDR3 that could potentially pair; 1 C unpaired, a single extra cysteine in CDR3; No C, no noncanonical cysteines found. VH/VL interface residues are marked with a *. Boxes define CDR1, CDR2 and CD3 regions.

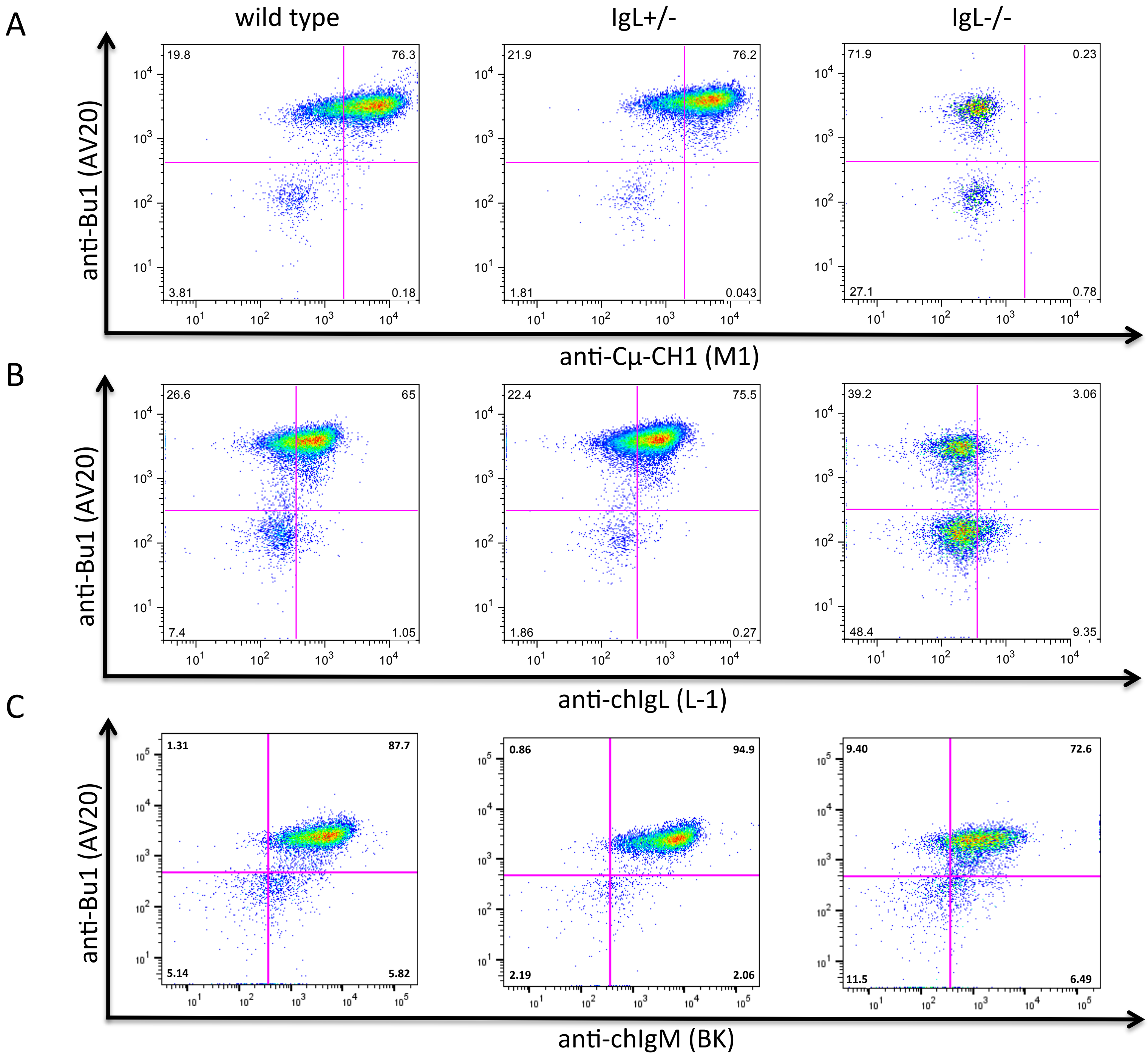

Supporting Information 4. B cells of IgL$^{-/-}$ chickens express immunoglobulin C*μ* on the cell surface. Bursas of one day-old wild type, IgL$^{+/-}$ and IgL$^{-/-}$ chickens were disrupted and leukocytes were isolated by Ficoll density gradient centrifugation. B cells were labeled with anti-chBu1-Alexa-647 (AV20) and (A), a monoclonal anti-C*μ*-CH1-FITC (M1) or (B), anti-chIgL-PE (L-1). For (C), cells were labeled with a monoclonal anti-chIgM antibody that does not bind CH1 (BK), followed by goat-anti-mouse IgG-APC. The cells were then blocked with mouse serum and labeled with Bu1-FITC (AV20). Cells were analyzed by flow cytometry. One representative plot out of at least four analyzed birds per genotype is shown.

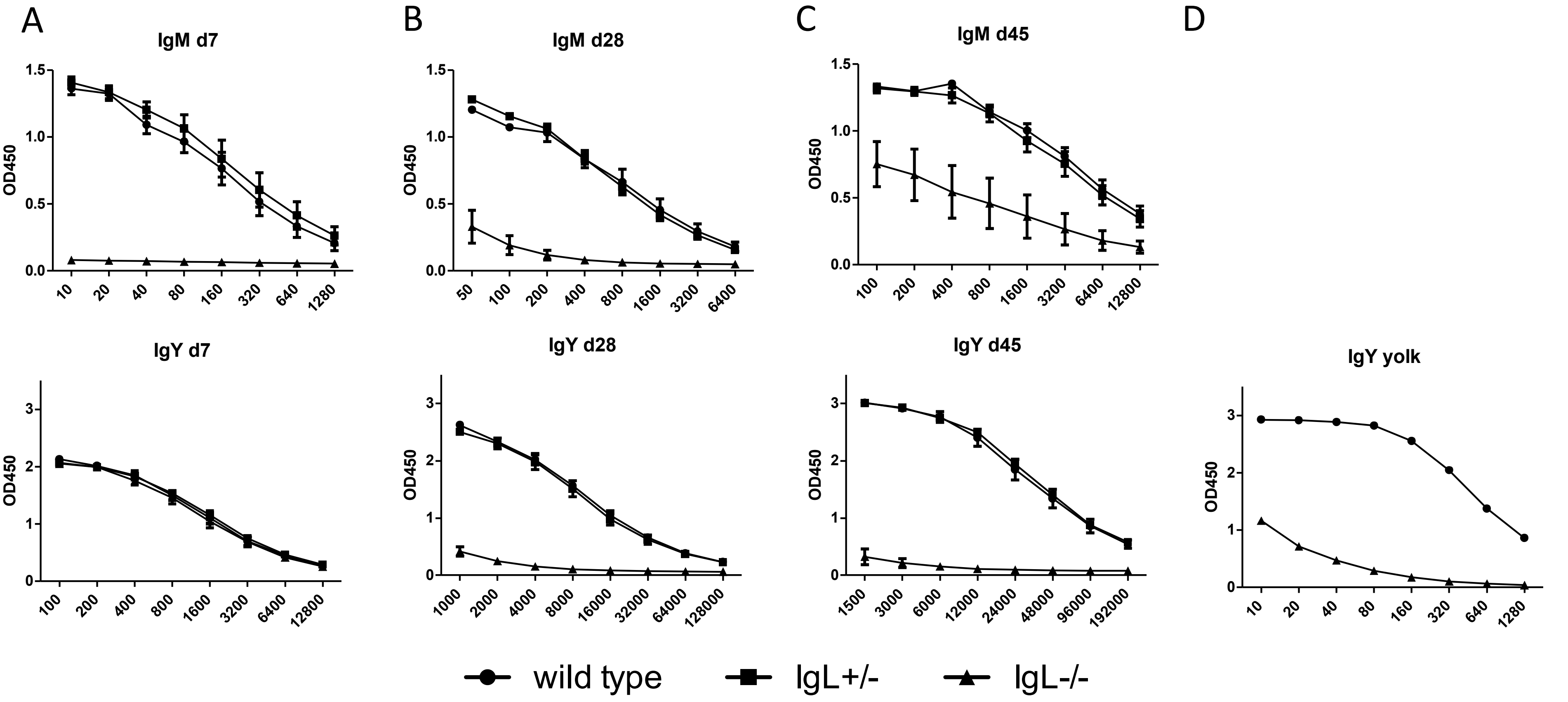

Supporting Information 5. IgL$^{-/-}$ chickens produce IgM and IgY.
Plasma samples from 7 (A), 28 (B) and 45 (C) day old chickens were analyzed by ELISA for total IgM and IgY levels. At day 7, the IgY signal is mainly maternally derived, which persists for over one week after hatch. Plates were coated with polyclonal anti-chIgM or polyclonal anti-chIgY, and detected with polyclonal anti-chIgM-HRP or polyclonal anti-chIgY-HRP. Yolk samples from eggs of six month old hens were checked for the presence of IgY (D). Mean and SEM of at least four birds per group are shown.

V region | CH2

VH | D | JH

```
SRDNGQSTVRLQLNNLRAEDTATYFCAKTT--CTGCSGPYAG---EIDAWGHGAEVIVSS|GPPIPTPLFVTMHP
SRDNGQSTLRLQLNNLRAEDTGTYYCARSSNSGYYCGPYGDC----IDAWGHGTEVIVSS|GPPIPTPLFVTMHP
SRDNGQSTVRLQLNNLRAEDTGTYYCARDF--GSSCGSAAYC----IDAWGHGTEVIVSS|GPPIPTPLFVTMHP
SRDNGQSTVRLQLNNLRAEDTATYYCARSP-GGYCCGPHDDS----MDAWGHGTEVIVSS|GPPIPTPLFVTMHP
SRDNGQSTVRLQLNNLRAEDTGAYYCAKGA--CSGCSSGFCY---SIDTWGHGTEVIVSS|GPPIPTPLFVTMHP
SKDNGQSTVRLQLNNLRAEDTGTYYCAKPADVGSAYGGSYRVGLIDIDARGHGTEVIVSS|GPPIPTPLFVTMHP
SRDNGQSTVRLQLNNLRAEDTATYYCTRST--GSGCTSDCAGGFIDIDAWGHGTEVIVSS|GPPIPTPLFVTMHP
SRDKGQSTVRLQLNNLRAEDTGTYYCAKAG--CD--SGAGVG---EIDGWGHGTEVIVSS|GPPIPTPLFVTMHP
SRDNGQSTVRLQLNNLRAEDTGTYYCAKDAYS-YGGGGNYEAG--CIDAWGHGTEVIVSS|GPPIPTPLFVTMHP
```

Supporting Information 6. IgM transcripts are spliced from the V region to the CH2 exon.
cDNA from day 28 PBMCs of IgL$^{-/-}$ birds was amplified as in Figure 3c and TA-cloned using primers in the VH and CH2 exons. Of 9 sequences obtained, all showed the same in-frame splicing event from the end of JH directly to CH2 (solid vertical line), with a deletion of CH1. Sequencing of PCR products amplified from genomic DNA (not shown) confirmed the intron-exon borders of the CH2 exon. As shown in the figure, the CH2 domain does not contain the 4 amino acids NGIP that were originally reported at positions 29-32.

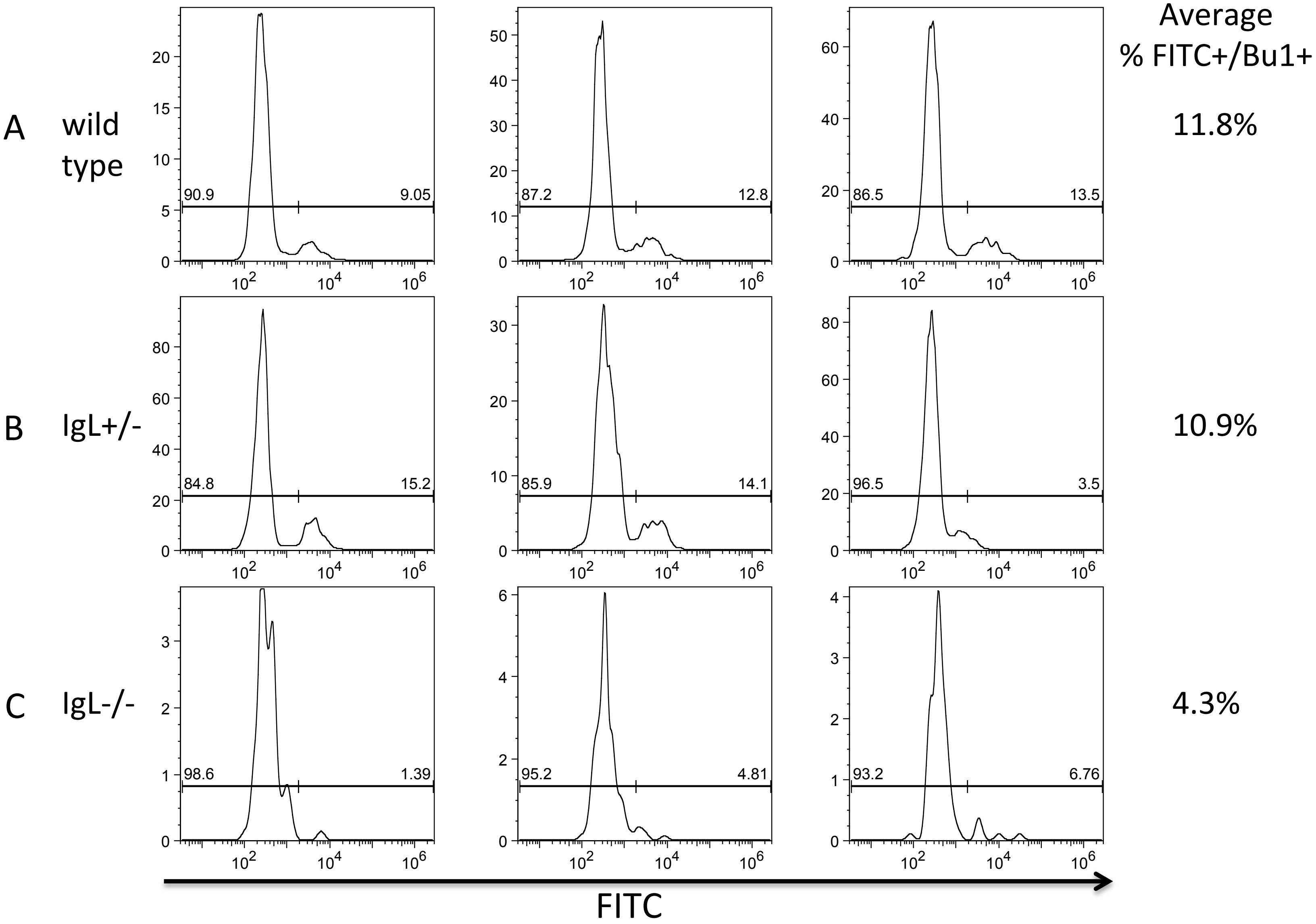

Supporting Information 7. B cells emigrate from the Bursa to the periphery in IgL$^{-/-}$ chicken.
100μl FITC solution (5mg/ml) was applied to the anal lips of 6 day old wild type (A), IgL$^{+/-}$ (B) and IgL$^{-/-}$ (C) chickens, and anal sucking movement took up the FITC solution. Ten hours after application PBMCs were isolated by Ficoll density gradient centrifugation and stained for B cells (anti-Bu1(AV20)). B cells and FITC-labeled cells were analyzed by flow cytometry. Three birds per genotype are displayed. Each graph shows a histogram of the Bu1^{+} cells, a small percentage of which are also FITC^{+}. The average % FITC for each genotype is listed.
