# Supplementary material for: Expression of heavy chain‐only antibodies can support B‐cell development in light chain knockout chickens
Source: Eur J Immunol. 2016 Aug 2;46(9):2137–48. doi: 10.1002/eji.201546171 (PMC5113765; doi:10.1002/eji.201546171)
Supplement: Supplementary file 2 — Peer Review Correspondence [file EJI-46-2137-s002.pdf]

European Journal of Immunology

Peer review correspondence

Expression of heavy chain-only antibodies can support B-cell development in light chain knockout chickens

Benjamin Schusser, Ellen J. Collarini, Darlene Pedersen, Henry Yi, Kathryn Ching, Shelley Izquierdo, Theresa Thoma, Sarah Lettmann, Bernd Kaspers, Robert J. Etches, Marie-Cecile van de Lavoir, William Harriman and Philip A. Leighton

Corresponding author: Philip A. Leighton, Crystal Bioscience Inc., Emeryville, California, United States

---

Review Timeline:	Submission date:	3 November 2015
	First editorial decision:	8 December 2015
	First revision received:	6 April 2016
	Second editorial decision:	4 May 2016
	Second revision received:	20 May 2016
	Accepted:	06 July 2016

Handling Executive Committee members: Prof. Hans-Martin Jäck

Please note that the correspondence below does not include the standard editorial instructions regarding preparation and submission of revised manuscripts, only the scientific revisions requested and addressed.

First Editorial Decision – 8 December 2015

Dear Dr. Leighton,

Manuscript ID eji.201546171 entitled "Expression of heavy chain-only antibodies supports B cell development in light chain knockout chickens" which you submitted to the European Journal of Immunology has been reviewed. The comments of the referees are included at the bottom of this letter.

You will see that the referees were in general enthusiastic about your submission and a common concern aside from the scientific ones was that the presentation and description of data could be optimised to be more informative and logical, and controls could either be included or better described. Therefore a revised version of your manuscript that takes into account the comments of the referees will be reconsidered for publication. Should you disagree with any of the referees' concerns, you should address

this in your point-by-point response and provide solid scientific reasons for why you will not make the requested changes.

You should also pay close attention to the editorial comments included below. *In particular, please edit your figure legends to follow Journal standards as outlined in the editorial comments. Failure to do this will result in delays in the re-review process.*

Please note that submitting a revision of your manuscript does not guarantee eventual acceptance, and that your revision will be re-reviewed by the referees before a decision is rendered.

If the revision of the paper is expected to take more than three months, please inform the editorial office. Revisions taking longer than six months may be assessed by new referees to ensure the relevance and timeliness of the data.

Once again, thank you for submitting your manuscript to European Journal of Immunology and we look forward to receiving your revision.

Yours sincerely,
Karen Chu

On behalf of Prof. Hans-Martin Jack

Dr. Karen Chu
Editorial Office
European Journal of Immunology
e-mail: ejied@wiley.com
www.eji-journal.eu

****

Reviewer: 1

Comments to the Author
This paper is interesting both from practical and theoretical points of view. Heavy-chain-only antibodies in a species that does not have the need for a surrogate light chain is an interesting “variation on the theme”, even if it is an artificial variation! In addition one can draw comparisons with mouse, camel and shark similar antibodies.

European Journal of Immunology

I have a few questions concerning the western blot of fig3
We need to know better the conditions. Clearly this section requires more explanations. From the moment the results are a little “bizarre” (this extra ca 90KD band), we need to know more about the conditions. Specificity control for the polyclonal? Reducing agent? Absorptions that were done etc Possible cross reactions? Percentage of the gel? More precise MW markers (to estimate better the strange so called 90 KD extra band in the IgA lanes.)
We need some potential explanation for this band.
It would not hurt to see much more of the western blot gel in the figure.

In order to better profit from fig supp 3 It might be useful to quote the paper by Reynaud et al, where the authors deal with the DH-regions , that are not available in genbank. (unless I miss something In which case it would be good tom indicate a reference in this paper.

Cell. 1989 Oct 6;59(1):171-83.
Somatic hyperconversion diversifies the single Vh gene of the chicken with a high incidence in the D region.
Reynaud CA1, Dahan A, Anquez V, Weill JC.

To better integrate the importance of the light chain minus antibodies in the vertebrate context, it would be useful in addition to the comparison with mouse to discuss the work done in shark. This paper was actually the first description of a natural H-chain only antibodies.
Nature. 1995 Mar 9;374(6518):168-73.
A new antigen receptor gene family that undergoes rearrangement and extensive somatic diversification in sharks.
Greenberg AS1, Avila D, Hughes M, Hughes A, McKinney EC, Flajnik MF.

Later a paper on convergence between shark and camel was published and In this context mention perhaps that the chicken heavy-chain-only antibodies do not contain an extra cystein in the cdr1, like camels and shark (although the D region encodes some.)

Proc Natl Acad Sci U S A. 1998 Sep 29; 95(20): 11804–11809.
Structural analysis of the nurse shark (new) antigen receptor (NAR): Molecular convergence of NAR and unusual mammalian immunoglobulins
Kenneth H. Roux,* Andrew S. Greenberg,† Lesley Greene,‡ Lioudmila Strelets,* David Avila,§ E. Churchill McKinney,† and Martin F. Flajnik†¶

Ref 27 is not a reference to camel as one is induce to believe by the bracket 26-28, but a paper on the possible usage of framework residues shared among species in order to humanize mouse antibodies.

European Journal of Immunology

It would be interesting to have data on the quality of the responses that have been observed: affinity, specificity of the antibodies. Obviously that was not the purpose of this paper but an opening on these issues would be welcome this paper does not have only a technical interest. The type of repertoire encountered in these animals might be quite surprising and worth looking at. Repertoire selection

Given the long time necessary to see the heavy-chain-only antibodies appear, one can speculate that the sequences seen in fig supp 3 are the product of a heavy selection hence the large difference with the references sequences yet no selection for H chains with an extra C close to o0r in CDR1 that would pair with the cdr3’s one, like in the camel and the shark H-only antibodies.

Reviewer: 2

Comments to the Author

This is an interesting study reporting the generation and phenotypic characterization of Ig Light Chain knock-out chickens (IgL-/-). This study follows an earlier study by the same group (PNAS 110:20170-5, 2013) on IgH-/- chickens. The results of the present study reveal that IgL-/- chicken have a strong reduction in the size of the Bursa, in the relative number of B cells in PBMC and spleen, and in plasma Ig levels. Notwithstanding, a small population of B cells could clearly be detected in spleen and Bursa, as well as very low levels of IgM, IgY and IgA in plasma. Western Blot and RT-PCR analyses indicate lack of the CH1 domain in IgM as a possible explanation for the residual production of heavy chain only IgM antibodies. This is reminiscent of the naturally occurring heavy chain only antibodies produced by camelids. Intriguingly, the residual IgA antibodies seem to retain the CH1 domain.

The data are of interest. However, many of the figures and parts of the text seem to have been assembled rather carelessly. Numerous mistakes unduly complicate understanding. Some controls are missing. The paper would benefit with the inclusion of controls, additional evidence, and a general overhaul of text and figures.

Major concerns:

1) The interpretation that IgL-/- chicken can produce intact IgA is interesting and should be tested experimentally, e.g. by PCR-amplifying and sequencing the VH-CH2 fragment for IgA. Similarly, deletion of CH1 from IgY sould be verified by PCR amplification and sequencing (as shown for IgM in Fig. 2 and Supp. 6).

European Journal of Immunology

2) The hypothesis that the CH1 domain is lacking because of splice site mutations is intersting and should be tested experimentally. PCR amplify the CH1 exon with flanking intronic sequences from genomic DNA of peripheral blood leukcytes (i.e. as done for the rearranged VH-D-J in Supp. 3.

3) The authors should determine whether the eggs of IgL-/- chicken contain CH1-deficient IgY antibodies.

4) The introduction contains a lot of superfluous information, e.g. Nobel prizes for ASV and RT, Salmonella pullorum, Avian Leukosis Virus and chicken in food supply. In contrast, a paragraph on naturally occurring heavy chain antibodies in camelids is missing.

5a) The FACS analyses using monoclonal anti-Bu1 and polyclonal anti-IgM that are used to claim that residual B cells display surface IgM are not convincing. Indirect staining with two polyclonal antibodies is always problematic, and should be corroborated with appropriate controls. Fig. 2d shows only percentages (60% of a small subset (< 2%) of total lymphocytes. Representative dot plots of PBMC from WT, IgL-/- and IgH-/- should be shown.

b) The corresponding FACS plots of cells from Bursa shown in Supp. Fig. 4c look strange: the density of dots does not correspond to those in a) and b). The slanted slope of the dot plots suggests a linear correlation of cell surface levels of Bu1 vs IgM? This looks like unspecific reactivity of the secondary antibody.

c) The corresponding materials and methods section is erroneous: the two antibodies used for co-staining carry the same fluorochrome: goat anti-chicken-IgM > donkey-anti-goat IgG Alexa647, ms anti-Bu-1 Alexa647

6) The capacity of IgL-/- chickens to produce antigen-specific antibodies in responses to immunization should be verified for at least two distinct antigens (e.g. BSA in addition to KLH). Moreover, analyses of antigen-specific responses should include appropriate controls (analyses of pre-immune serum, lack of reactivity of immune sera to control antigen).

7) The description of the spleen histology in the text does not match the data shown in Fig. 6b. The text claims that no differences in B and T cell stainings were detected between WT and IgL-/- at day 45, while the stainings shown in Fig. 6b top panels look rather different.

8) In the last paragraph of the results section on emigration of B cells from the bursa (p.6 line 44:), the % of Bu1/FITC double positive cells given in the text do not at all match the data shown in the corresponding figure (Fig. S7): 17.5%, 13.5% and 18.1% vs. 9/12.8/13.5, 15.2/14.1/3.5, 1.4, 4.8, 6.8.

European Journal of Immunology

Minor points

1) p.4 line 51: The statement that 45d after hatch, IgM, IgY and IgA are clearly produced in IgL-/- birds is misleading. The authors should point out that the levels of these Abs in IgL-/- birds are at least 100 fold lower than those in WT birds.

2) p.5 line 40: show data that no KLH antibodies were detectable in any group prior to immunization

3) Figs 2-6 and Supp. Figs 2, 4, 5, 6: the labels wildtype, heterozygous, homozygous are potentially confusing (wildtype animals are also homozygous). I suggest to replace these by WT, IgL +/-, IgL -/-

4) Fig. 2a: Show representative FACS plots of WT vs. IgL-/- PBMC and splenic B cells vs. T cells (Bu1 vs. TCR2/3) gated on lymphocytes. The Y axes in should indicate the reference cell type (e.g. % of lymphocytes, or % of B cells).

5) Fig. 2b: Include two or three lanes with serum samples from IgH-/- chickens for comparison.

6) Fig. 3a) This figure would be much easier to understand if the authors included a side by side comparison of ELISAs performed with anti-CH1 vs. anti-IgM, e.g. as shown in panel b) of Supp Fig. 5.

7) Fig. 4: negative controls are missing, i.e. titration of pre-immuneserum vs. KLH and of d12 immune serum vs. an irrelevant protein, e.g. BSA

8) Fig. 5: indicate cortex/medulla by arrows

9) Fig. 6: indicate dark and light zones by arrows. The arrangement of panels in a) and b) is confusing, i.e. the four lower panels correspond in terms of time and antibodies used for staining, while the upper panels do not. Include stainings of d1 spleen with chIgM-M1 and Polyclonal achIgM, and of d45 Bursa with BU1 and TCR1+TCR2.

10) Supp. 3: The alignments of the amino acid sequences of the CDR2 and CDR3 regions appear awkward because dashes for missing amino acids are placed in various positions. It would be much clearer if the dashes for missing amino acids were placed at the end of the CDR2 or CDR3, respectively. The authors should comment on sets and families of clones that are evidently derived from the same B cell clone.

11) Supp 5: The same X-axis scale is used for IgA d7, d28, and d45; but not for IgM and IgY. This complicates understanding of the results for IgM and IgY. The data for different days should be presented with the same X-axis scale also for IgM and IgY.

European Journal of Immunology

12) methods p. 9, line 27: M1 is listed twice,

13) reference 27 does not deal with camelid antibodies. Indeed it was published before the seminal paper on camelid Abs by Hamers et al in 1993 in Nature 363.

Reviewer: 3

Comments to the Author
Overall this is an interesting paper describing the phenotype of chickens which cannot express L chain. The findings are surprising and novel – one would have predicted that chickens that cannot synthesize L chains would be completely blocked in B cell development. That is not the case. However, there are some small issues that could be addressed to improve the paper.

The first paragraph of the Introduction, while interesting is in most part not particularly relevant to the subject of this paper.

While it may seem obvious what PGCs are, they should be defined.
What Bu1 recognizes should be defined the first time the marker is introduced (Fig 2).

It is unclear exactly what the authors mean when they say that "expression of the heavy chain protein is being selected"

References should be given for papers describing the role of BiP in ER retention of Igs.

Ig levels (Fig S5b and 4b) would be better if expressed as µg (or mg)/ml in a table, possibly with the levels of individual birds noted.

More extensive analysis of IgA production in the IgL-/- should be made. What is the composition of the larger molecular weight band seen in the Western blot of IgA (Fig 3b)? The Discussion should include some discussion of why alpha heavy chains can be secreted in the absence of L chain.

It is unclear exactly what is being shown in figure Suppl. 7. It should be more clearly labeled. Do the rows or columns represent the 3 different birds. Were different parameters analyzed for the different birds?

European Journal of Immunology

First revision – authors' response – 6 April 2016

Reviewer: 1

Comments to the Author

This paper is interesting both from practical and theoretical points of view. Heavy-chain-only antibodies in a species that does not have the need for a surrogate light chain is an interesting "variation on the theme", even if it is an artificial variation! In addition one can draw comparisons with mouse, camel and shark similar antibodies.

I have a few questions concerning the western blot of fig3

We need to know better the conditions. Clearly this section requires more explanations. From the moment the results are a little "bizarre" (this extra ca 90KD band), we need to know more about the conditions. Specificity control for the polyclonal? Reducing agent? Absorptions that were done etc Possible cross reactions? Percentage of the gel? More precise MW markers (to estimate better the strange so called 90 KD extra band in the IgA lanes.)

We need some potential explanation for this band.

It would not hurt to see much more of the western blot gel in the figure.

Author response: We repeated the IgA Westerns with different antibodies and further controls and have now come to the conclusion that there is little or no IgA present in the IgL-/- birds and if there is any, it is below our level of detection. All of the anti-IgA antibodies we tried (two monoclonals and one polyclonal) showed high levels of background, including extra bands such as the 90 kD band which we found were present in all samples including wild type and heavy chain knockouts. The Western blot now in Fig 3 was done using the antibody with the best signal to noise, along with the heavy chain KO control (JH-/-) to show that the background bands are non-specific. We believe that the signal observed in the IgA ELISA at day 45 was also background, since heavy chain knockout samples showed a similar signal, so we have removed the IgA ELISAs from Supp 5. We further investigated IgA by performing an immunoprecipitation using the same antibodies used for the ELISA, and found no IgA present in the IgL-/- serum.

In order to better profit from fig supp 3 It might be useful to quote the paper by Reynaud et al, where the authors deal with the DH-regions , that are not available in genbank. (unless I miss something In which case it would be good tom indicate a reference in this paper.

Cell. 1989 Oct 6;59(1):171-83.

Somatic hyperconversion diversifies the single Vh gene of the chicken with a high incidence in the D region.

Reynaud CA1, Dahan A, Anquez V, Weill JC.

European Journal of Immunology

Author response: We have included the reference from Reynaud on the DH-regions, and a sentence in the Discussion regarding the cysteines encoded by the DH regions.

To better integrate the importance of the light chain minus antibodies in the vertebrate context, it would be useful in addition to the comparison with mouse to discuss the work done in shark. This paper was actually the first description of a natural H-chain only antibodies.
Nature. 1995 Mar 9;374(6518):168-73.
A new antigen receptor gene family that undergoes rearrangement and extensive somatic diversification in sharks.
Greenberg AS1, Avila D, Hughes M, Hughes A, McKinney EC, Flajnik MF.

Later a paper on convergence between shark and camel was published and In this context mention perhaps that the chicken heavy-chain-only antibodies do not contain an extra cystein in the cdr1, like camels and shark (although the D region encodes some.)

Proc Natl Acad Sci U S A. 1998 Sep 29; 95(20): 11804–11809.
Structural analysis of the nurse shark (new) antigen receptor (NAR): Molecular convergence of NAR and unusual mammalian immunoglobulins
Kenneth H. Roux,* Andrew S. Greenberg,† Lesley Greene,‡ Lioudmila Strelets,* David Avila,§ E. Churchill McKinney,† and Martin F. Flajnik†¶
Author response: Thank you for pointing out our accidental omission of the data from shark. We have added these references and discussion of shark antibodies into the paper.

Ref 27 is not a reference to camel as one is induce to believe by the bracket 26-28, but a paper on the possible usage of framework residues shared among species in order to humanize mouse antibodies.
Author response: Ref 27 has been fixed.

It would be interesting to have data on the quality of the responses that have been observed: affinity, specificity of the antibodies. Obviously that was not the purpose of this paper but an opening on these issues would be welcome this paper does not have only a technical interest. The type of repertoire encountered in these animals might be quite surprising and worth looking at. Repertoire selection
Author response: We confirmed the specificity of the antisera by ELISA, showing that they are specific for the immunogen KLH and don't bind an unrelated protein (BSA). The BSA data are now included in Figure 4. The data indicate that the IgL -/- birds are capable of producing functional heavy-chain antibodies. We feel that making a panel of monoclonal antibodies would be beyond the scope of this paper, which is primarily to assess the phenotype of the knockout.

European Journal of Immunology

Given the long time necessary to see the heavy-chain-only antibodies appear, one can speculate that the sequences seen in fig supp 3 are the product of a heavy selection hence the large difference with the references sequences yet no selection for H chains with an extra C close to o0r in CDR1 that would pair with the cdr3's one, like in the camel and the shark H-only antibodies.

Reviewer: 2

Comments to the Author
This is an interesting study reporting the generation and phenotypic characterization of Ig Light Chain knock-out chickens (IgL-/-). This study follows an earlier study by the same group (PNAS 110:20170-5, 2013) on IgH-/- chickens. The results of the present study reveal that IgL-/- chicken have a strong reduction in the size of the Bursa, in the relative number of B cells in PBMC and spleen, and in plasma Ig levels. Notwithstanding, a small population of B cells could clearly be detected in spleen and Bursa, as well as very low levels of IgM, IgY and IgA in plasma. Western Blot and RT-PCR analyses indicate lack of the CH1 domain in IgM as a possible explanation for the residual production of heavy chain only IgM antibodies. This is reminiscent of the naturally occurring heavy chain only antibodies produced by camelids. Intriguingly, the residual IgA antibodies seem to retain the CH1 domain.

The data are of interest. However, many of the figures and parts of the text seem to have been assembled rather carelessly. Numerous mistakes unduly complicate understanding. Some controls are missing. The paper would benefit with the inclusion of controls, additional evidence, and a general overhaul of text and figures.

Major concerns:
1) The interpretation that IgL-/- chicken can produce intact IgA is interesting and should be tested experimentally, e.g. by PCR-amplifying and sequencing the VH-CH2 fragment for IgA. Similarly, deletion of CH1 from IgY sould be verified by PCR amplification and sequencing (as shown for IgM in Fig. 2 and Supp. 6).
Author response: For IgA, we now believe that our detection antibodies have high non-specific background and that specific IgA signal is not detected in the IgL-/- chickens. We repeated our experiments using control samples from heavy chain knockout birds (JH-/-), which showed essentially identical results as IgL-/- birds on Western and ELISA. We tried two monoclonals and one polyclonal against IgA and reached the same conclusion. Assuming that the JH-/- birds have no IgA, since they have no B cells and no IgM expression (as shown in our referenced paper), we believe that the bands on Western are non-specific, including the ~90kD band we originally observed. The Western blot now in Fig 3 was done using the antibody with the best signal to noise, along with the JH-/- control to show that the background bands are non-specific. The relatively high signal on the d45 ELISA that we originally

submitted was also non-specific, and so we have removed the IgA Elisas from Supp. Fig 5. We also performed an immunoprecipitation for IgA from serum of IgL-/- and wild type birds and did not detect any IgA in the knockouts. Thus we conclude that there is little, if any, IgA produced as it is below our level of detection. RT-PCR for IgA from PBMC came up blank, using primers in various Calpha domains. For IgY, unexpectedly, the RT-PCR amplification does not show a deletion of CH1, even though there is a band on the Western in some protein samples showing a reduced molecular weight for IgY. The results are now included in Fig. 3c. We interpret the results to mean that the vast majority of the mRNA is correctly spliced, potentially producing a full-length protein that cannot be secreted, and only a tiny amount of the mRNA is spliced to remove the CH1 domain. The text has been modified to reflect this.

2) The hypothesis that the CH1 domain is lacking because of splice site mutations is intersting and should be tested experimentally. PCR amplify the CH1 exon with flanking intronic sequences from genomic DNA of peripheral blood leukcytes (i.e. as done for the rearranged VH-D-J in Supp. 3.
Author response: Amplification of the CH1 exon from genomic DNA would not be feasible since the genomic sequence of the chicken heavy chain has not been completed and the intron sequence downstream of CH1 is unknown. We were able to obtain some sequence information around the CH2 exon. The genomic region around the V and J exons is known, which allowed us to amplify the rearranged VDJ exon for the data in Supp 3.

3) The authors should determine whether the eggs of IgL-/- chicken contain CH1-deficient IgY antibodies.
Author response: Low levels of IgY are deposited into the egg yolk of IgL-/- hens. The data are now included in Fig. Supp 5.

4) The introduction contains a lot of superfluous information, e.g. Nobel prizes for ASV and RT, Salmonella pullorum, Avian Leukosis Virus and chicken in food supply. In contrast, a paragraph on naturally occurring heavy chain antibodies in camelids is missing.
Author response: We have re-written the Introduction and removed the information mentioned above, and added information about naturally occurring heavy chain-only antibodies.

5a) The FACS analyses using monoclonal anti-Bu1 and polyclonal anti-IgM that are used to claim that residual B cells display surface IgM are not convincing. Indirect staining with two polyclonal antibodies is always problematic, and should be corroborated with appropriate controls. Fig. 2d shows only percentages (60% of a small subset (< 2%) of total lymphocytes. Representative dot plots of PBMC from WT, IgL-/- and IgH-/- should be shown.
Author response: See below.

b) The corresponding FACS plots of cells from Bursa shown in Supp. Fig. 4c look strange: the density of dots does not correspond to those in a) and b). The slanted slope of the dot plots suggests a linear

European Journal of Immunology

correlation of cell surface levels of Bu1 vs IgM? This looks like unspecific reactivity of the secondary antibody.

Author response: For items 5a) and 5b): We have repeated the experiment in Supp. Fig. 4c using a monoclonal antibody to IgM to avoid any non-specific cross-reactivities, and the results are essentially the same. Considering this, we do not think the PBMC results in Fig 2d, which were labeled the same way, will change significantly. The dot plots in Supp. Fig. 4 are representative of B cell labeling, even though they are on bursal cells rather than PBMCs. We do not feel that showing more dot plots will improve the manuscript.

c) The corresponding materials and methods section is erroneous: the two antibodies used for co-staining carry the same fluorochrome: goat anti-chicken-IgM > donkey-anti-goat IgG Alexa647, ms anti-Bu-1 Alexa647

Author response: The antibodies that were used for each experiment are now listed in the Figure legends to avoid any ambiguity.

6) The capacity of IgL-/- chickens to produce antigen-specific antibodies in responses to immunization should be verified for at least two distinct antigens (e.g. BSA in addition to KLH). Moreover, analyses of antigen-specific responses should include appropriate controls (analyses of pre-immune serum, lack of reactivity of immune sera to control antigen).

Author response: We now include a specificity ELISA of our immune sera showing lack of reactivity to a control protein (BSA). The data are in Fig. 4.

7) The description of the spleen histology in the text does not match the data shown in Fig. 6b. The text claims that no differences in B and T cell stainings were detected between WT and IgL-/- at day 45, while the stainings shown in Fig. 6b top panels look rather different.

Author response: The density of germinal centers in the knockout is lower, which explains why the pictures look slightly different in the top panels of Fig. 6B, but the structure of individual germinal centers is the same as in wild type. We have revised the wording in the text to clarify this point.

8) In the last paragraph of the results section on emigration of B cells from the bursa (p.6 line 44:), the % of Bu1/FITC double positive cells given in the text do not at all match the data shown in the corresponding figure (Fig. S7): 17.5%, 13.5% and 18.1% vs. 9/12.8/13.5, 15.2/14.1/3.5, 1.4, 4.8, 6.8.

Author response: The numbers given in the text were taken from a different calculation. Thank you for pointing out the error. We have made this figure easier to read and fixed the text and figure legend.

European Journal of Immunology

Minor points

1) p.4 line 51: The statement that 45d after hatch, IgM, IgY and IgA are clearly produced in IgL-/- birds is misleading. The authors should point out that the levels of these Abs in IgL-/- birds are at least 100 fold lower than those in WT birds.

Author response: We have revised the text to make it clear the antibody levels are much lower in the IgL-/- birds.

2) p.5 line 40: show data that no KLH antibodies were detectable in any group prior to immunization

Author response: We now show pre-immune ELISA in Fig. 4.

3) Figs 2-6 and Supp. Figs 2, 4, 5, 6: the labels wildtype, heterozygous, homozygous are potentially confusing (wildtype animals are also homozygous). I suggest to replace these by WT, IgL +/-, IgL -/-

Author response: This change has been made throughout the text and figures.

4) Fig. 2a: Show representative FACS plots of WT vs. IgL-/- PBMC and splenic B cells vs. T cells (Bu1 vs. TCR2/3) gated on lymphocytes. The Y axes in should indicate the reference cell type (e.g. % of lymphocytes, or % of B cells).

Author response: Supp. Fig 4 shows representative FACS dot plots for B cell labeling. Although these dot plots are for bursal cells, the same procedure and analysis was used for the PBMCs and spleen cells shown in Fig. 2; we do not think adding more FACS plots would improve the manuscript. The splenic B and T cells were labeled by taking 2 aliquots of cells and labeling each with a single antibody; the cell population was not double-labeled, so we cannot graph Bu1 vs. TCR2/3. The Y axes for all graphs in Fig. 2 have been re-labeled.

5) Fig. 2b: Include two or three lanes with serum samples from IgH-/- chickens for comparison.

Author response: We assume the reviewer is referring to Fig3b; we have now included a JH-/- sample for the IgA blot.

6) Fig. 3a) This figure would be much easier to understand if the authors included a side by side comparison of ELISAs performed with anti-CH1 vs. anti-IgM, e.g. as shown in panel b) of Supp Fig. 5.

Author response: We now include both the anti-CH1 and anti-IgM ELISAs in Fig 3.

7) Fig. 4: negative controls are missing, i.e. titration of pre-immuneserum vs. KLH and of d12 immune serum vs. an irrelevant protein, e.g. BSA

Author response: We now include an ELISA to BSA for the KLH-immune serum.

8) Fig. 5: indicate cortex/medulla by arrows

Author response: Cortex and medulla are now labeled.

European Journal of Immunology

9) Fig. 6: indicate dark and light zones by arrows. The arrangement of panels in a) and b) is confusing, i.e. the four lower panels correspond in terms of time and antibodies used for staining, while the upper panels do not. Include stainings of d1 spleen with chIgM-M1 and Polyclonal achIgM, and of d45 Bursa with BU1 and TCR1+TCR2.

Author response: Dark and light zones are now labeled. Panels 6A and 6B are intended to be independent, panel A for bursa and panel B for spleen. The staining and time points happen to correspond for the lower four panels by coincidence. We have labeled the panels more clearly with the time point for each. In the bursa, we have focused on B-cell specific staining and haven't done T-cell staining with TCR1/TCR2. We did not analyze spleen at day 1 since that is very early in spleen development, and very few B cells would have normally migrated there by that time.

10) Supp. 3: The alignments of the amino acid sequences of the CDR2 and CDR3 regions appear awkward because dashes for missing amino acids are placed in various positions. It would be much clearer if the dashes for missing amino acids were placed at the end of the CDR2 or CDR3, respectively. The authors should comment on sets and families of clones that are evidently derived from the same B cell clone.

Author response: The alignment has been re-done to try to make the CDR sequences more clear. However, the dashes for missing amino acids are placed by the alignment program where there is a deletion in comparison to the reference, so they fall where they occur. The sequences were grouped into families of clones and we have included some new analysis of the sequences into the Results and Discussion sections.

11) Supp 5: The same X-axis scale is used for IgA d7, d28, and d45; but not for IgM and IgY. This complicates understanding of the results for IgM and IgY. The data for different days should be presented with the same X-axis scale also for IgM and IgY.

Author response: The dilution curves are a relative measure of the Ig present in the different genotypes at each time point. Since the level of Ig is increasing as the birds get older, it is necessary to use higher dilutions at older ages so that the dilution curve can be visualized.

12) methods p. 9, line 27: M1 is listed twice,

Author response: We had listed both the conjugated and unconjugated; we've removed the duplicates.

13) reference 27 does not deal with camelid antibodies. Indeed it was published before the seminal paper on camelid Abs by Hamers et al in 1993 in Nature 363.

Author response: Reference 27 has been fixed.

European Journal of Immunology

Reviewer: 3

Comments to the Author

Overall this is an interesting paper describing the phenotype of chickens which cannot express L chain. The findings are surprising and novel – one would have predicted that chickens that cannot synthesize L chains would be completely blocked in B cell development. That is not the case. However, there are some small issues that could be addressed to improve the paper.

The first paragraph of the Introduction, while interesting is in most part not particularly relevant to the subject of this paper.

Author response: We have extensively revised the Introduction.

While it may seem obvious what PGCs are, they should be defined.

What Bu1 recognizes should be defined the first time the marker is introduced (Fig 2).

Author response: We defined both of these terms.

It is unclear exactly what the authors mean when they say that “expression of the heavy chain protein is being selected”

Author response: We added the words “during development” to indicate we mean the process of selection that B cells undergo during development to eliminate cells that do not successfully express surface B cell receptor.

References should be given for papers describing the role of BiP in ER retention of Igs.

Author response: We have added these references.

Ig levels (Fig S5b and 4b) would be better if expressed as µg (or mg)/ml in a table, possibly with the levels of individual birds noted.

Author response: The relative levels of Ig in the mutants is much lower than in controls, which is the relevant information. Quantitation of the levels would require purchasing expensive kits to obtain purified Ig standards.

More extensive analysis of IgA production in the IgL-/- should be made. What is the composition of the larger molecular weight band seen in the Western blot of IgA (Fig 3b)? The Discussion should include some discussion of why alpha heavy chains can be secreted in the absence of L chain.

Author response: As discussed above, we have done more analysis, using different detection antibodies and including JH-/- samples, and now believe that we are not detecting IgA and that little or no IgA is produced in the IgL-/- birds. The previously shown larger molecular weight band is also detected in JH-/- samples and we conclude it is non-specific.

European Journal of Immunology

It is unclear exactly what is being shown in figure Suppl. 7. It should be more clearly labeled. Do the rows or columns represent the 3 different birds. Were different parameters analyzed for the different birds?
Author response: We have made this figure easier to read and re-written the text and figure legend.

Second Editorial Decision – 4 May 2016

Dear Dr. Leighton,

Thank you for submitting your revised manuscript ID eji.201546171.R1 entitled "Expression of heavy chain-only antibodies can support B cell development in light chain knockout chickens" to the European Journal of Immunology. Your manuscript has been re-reviewed and the comments of the referees are included at the bottom of this letter.

Although the referees have recommended publication, some revisions to your manuscript have been requested by referee 3. Therefore, I invite you to respond to the comments of this referee, as well as the editorial comments in the attached edited text file, and revise your manuscript accordingly.

You should also pay close attention to the editorial comments included below. *In particular, please edit your figure legends to follow Journal standards as outlined in the editorial comments. Failure to do this will result in delays in the re-review process.*

If the revision of the paper is expected to take more than three months, please inform the editorial office. Revisions taking longer than six months may be assessed by new referees to ensure the relevance and timeliness of the data.

Once again, thank you for submitting your manuscript to European Journal of Immunology. We look forward to receiving your revision.

Yours sincerely,
Karen Chu

on behalf of Prof. Hans-Martin Jack

Dr. Karen Chu
Editorial Office
European Journal of Immunology
e-mail: ejied@wiley.com

European Journal of Immunology

www.eji-journal.eu

****

Reviewer: 1

Comments to the Author

Thank you of following the suggestions.

Reviewer: 2

Comments to the Author

The authors have addressed all issues adequately.

Reviewer: 3

Comments to the Author

1. Does not adequately describe the data shown in Figure S2b where significant differences in bursa weight between groups are indicated. This needs to be commented on and explained.

2. The bar graph presentation of the staining data in Figure 2 does not convincingly show Bu+ B cells for any except for the day 45 spleen. The PBMC data for day 35 (2d) need to be better described. What are the significant differences indicating – for only wild-type or for heterogyzous animals? Need a better description.

3. Fig S4 - Is there an explanation for the high % of anti-Bu1 negative cells seen in the bursa stained with anti-ChIgL but not the others.

4. Figure s5 -- sholdl emphasize that the levels of IgM and IgY in the L- lines are VERY much lower – generally less than 1% of the levels in wild-type animals.

5. In Figure 3 if there is no deletion in IgY (3c) why is the protein small (3b). This is mentioned in the discussion, but not adequately.

6. A better description of Figure s7 is needed.

7. In the Discussion a clearer discussion of the origin of adjacent Cs is needed.

8. The rational for why additional Cs would be needed should to be clarified.

Peer review correspondence

European Journal of Immunology

Second revision – authors' response – 20 May 2016

Responses to Reviewer 3:

1. Does not adequately describe the data shown in Figure S2b where significant differences in bursa weight between groups are indicated. This needs to be commented on and explained.
We removed the weight data from embryonic day 18. The data from day 1 are sufficient to make the point, as it is only a few days later (hatch is at 21 days).

2. The bar graph presentation of the staining data in Figure 2 does not convincingly show Bu+ B cells for any except for the day 45 spleen. The PBMC data for day 35 (2d) need to be better described. What are the significant differences indicating – for only wild-type or for heterogyzous animals? Need a better description.
We tried to clarify in the text that the numbers of Bu1+ cells are low in the knockout, so the bars are very small in the graphs in Fig 2 for PBMC. We have re-written the description of 2d in the Results section and the Figure Legend to improve clarity.

3. Fig S4 - Is there an explanation for the high % of anti-Bu1 negative cells seen in the bursa stained with anti-ChIgL but not the others.
The percentage of Bu1-negative cells is generally higher in the homozygous knock-out than in the controls; the differences seen here can be explained by individual variation. This does not change the main point that there are Bu1-positive cells that are light chain negative and heavy chain positive.

4. Figure s5 -- sholdl emphasize that the levels of IgM and IgY in the L- lines are VERY much lower – generally less than 1% of the levels in wild-type animals.
We do already state that the levels are much lower than wild-type, in several places.

5. In Figure 3 if there is no deletion in IgY (3c) why is the protein small (3b). This is mentioned in the discussion, but not adequately.
We feel the statement in the discussion is sufficient -- we simply added the word "unexpectedly" since the RT-PCR and protein data are not in agreement for IgY, but we don't really have an explanation at this point.

6. A better description of Figure s7 is needed.
We have re-written the description of Supplemental Information Fig 7 in the Results and in the Figure Legend.

7. In the Discussion a clearer discussion of the origin of adjacent Cs is needed.

European Journal of Immunology

We have stated that the pattern of adjacent Cs is potentially from germline D segments. We have re-written this section to add more detail and clarify our results in the context of previous data.

8. The rational for why additional Cs would be needed should to be clarified.
We have added a statement regarding stabilization of CDR-H3 loops as to why Cs might be paired.

Third Editorial Decision – 8 June 2016

Dear Dr. Leighton,

It is a pleasure to provisionally accept your manuscript entitled "Expression of heavy chain-only antibodies can support B cell development in light chain knockout chickens" for publication in the European Journal of Immunology. For final acceptance, please follow the instructions below and return the requested items as soon as possible as we cannot process your manuscript further until all items listed below are dealt with.

Please note that EJI articles are now published online a few days after final acceptance (see Accepted Articles: http://onlinelibrary.wiley.com/journal/10.1002/(ISSN)1521-4141/accepted). The files used for the Accepted Articles are the final files and information supplied by you in Manuscript Central. You should therefore check that all the information (including author names) is correct as changes will NOT be permitted until the proofs stage.

We look forward to hearing from you and thank you for submitting your manuscript to the European Journal of Immunology.

Yours sincerely,
Karen Chu

on behalf of Prof. Hans-Martin Jack

Dr. Karen Chu
Editorial Office
European Journal of Immunology
e-mail: ejied@wiley.com
www.eji-journal.eu
